# First Genome-Wide Association Study in an Australian Aboriginal Population Provides Insights into Genetic Risk Factors for Body Mass Index and Type 2 Diabetes

**DOI:** 10.1371/journal.pone.0119333

**Published:** 2015-03-11

**Authors:** Denise Anderson, Heather J. Cordell, Michaela Fakiola, Richard W. Francis, Genevieve Syn, Elizabeth S. H. Scaman, Elizabeth Davis, Simon J. Miles, Toby McLeay, Sarra E. Jamieson, Jenefer M. Blackwell

**Affiliations:** 1 Telethon Kids Institute, The University of Western Australia, Subiaco, Western Australia, 6008, Australia; 2 Institute of Genetic Medicine, Newcastle University, Newcastle upon Tyne, NE1 3BZ, United Kingdom; 3 Cambridge Institute for Medical Research, Department of Medicine, and Department of Pathology, University of Cambridge, Cambridge, United Kingdom; 4 Department of Endocrinology and Diabetes, Princess Margaret Hospital for Children, Subiaco, Western Australia, 6008, Australia; 5 Ngangganawili Aboriginal Health Service, Wiluna, Western Australia, 6646, Australia; Graduate School of Medicine, University of the Ryukyus, JAPAN

## Abstract

A body mass index (BMI) >22kg/m^2^ is a risk factor for type 2 diabetes (T2D) in Aboriginal Australians. To identify loci associated with BMI and T2D we undertook a genome-wide association study using 1,075,436 quality-controlled single nucleotide polymorphisms (SNPs) genotyped (Illumina 2.5M Duo Beadchip) in 402 individuals in extended pedigrees from a Western Australian Aboriginal community. Imputation using the thousand genomes (1000G) reference panel extended the analysis to 6,724,284 post quality-control autosomal SNPs. No associations achieved genome-wide significance, commonly accepted as P<5x10^-8^. Nevertheless, genes/pathways in common with other ethnicities were identified despite the arrival of Aboriginal people in Australia >45,000 years ago. The top hit (rs10868204 *P*_genotyped_ = 1.50x10^-6^; rs11140653 P_imputed_1000G_ = 2.90x10^-7^) for BMI lies 5’ of *NTRK2*, the type 2 neurotrophic tyrosine kinase receptor for brain-derived neurotrophic factor (BDNF) that regulates energy balance downstream of melanocortin-4 receptor (MC4R). PIK3C2G (rs12816270 P_genotyped_ = 8.06x10^-6^; rs10841048 P_imputed_1000G_ = 6.28x10^-7^) was associated with BMI, but not with T2D as reported elsewhere. BMI also associated with *CNTNAP2* (rs6960319 P_genotyped_ = 4.65x10^-5^; rs13225016 P_imputed_1000G_ = 6.57x10^-5^), previously identified as the strongest gene-by-environment interaction for BMI in African-Americans. The top hit (rs11240074 P_genotyped_ = 5.59x10^-6^, P_imputed_1000G_ = 5.73x10^-6^) for T2D lies 5’ of *BCL9* that, along with *TCF7L2*, promotes beta-catenin’s transcriptional activity in the WNT signaling pathway. Additional hits occurred in genes affecting pancreatic (*KCNJ6*, *KCNA1*) and/or GABA (*GABRR1*, *KCNA1*) functions. Notable associations observed for genes previously identified at genome-wide significance in other populations included *MC4R* (P_genotyped_ = 4.49x10^-4^) for BMI and *IGF2BP2* P_imputed_1000G_ = 2.55x10^-6^) for T2D. Our results may provide novel functional leads in understanding disease pathogenesis in this Australian Aboriginal population.

## Introduction

Genome-wide association studies (GWAS) have been used with great success to identify genes associated with complex diseases [1], including obesity and type 2 diabetes (T2D) [2–6]. However, there are no published data on the use of this approach to study complex diseases in Aboriginal Australians. This is partly a reflection of the controversy surrounding genetic research in indigenous communities [7, 8], which has raised ethical concerns including a lack of benefit to community and diversion of attention and resources from non-genetic causes of health disparities and racism in health care [9–12]. Controversy relating to the Human Genetic Diversity Project has acted as a particular barrier to conducting genetic research in Australian Aboriginal communities [13], but there is now a strong lobby to bring the benefits of health-based genomic research to Australian Aboriginal populations [14] as there is for all ethnicities globally [8, 15]. T2D and associated pathologies are a major health problem in Indigenous Australians, and a body mass index (BMI) >22kg/m^2^ is a significant risk factor for T2D in Australian Aboriginal populations [16]. Here we present the first GWAS of BMI and T2D in a Western Australian Aboriginal population.

## Materials and Methods

### Study population

Subjects for the discovery GWAS were recruited from an Australian Aboriginal community of Martu ancestry [17, 18] at the edge of the Western Desert in Western Australia. A family-based study was proposed to take account of recent population history and shared ancestry. A memorandum of understanding (MoU) was established with the community, which included permission for access to clinical records held in a Communicare database at the local Aboriginal Health Service. An individual was classified as having T2D if the subject was: (1) diagnosed with T2D by a qualified physician; (2) on a prescribed drug treatment regimen for T2D; and (3) returned biochemical test results of a fasting plasma glucose level of at least 7 mmol/l in SI units based on criteria laid down by the World Health Organization (WHO) consultation group report [19]. Multiple height and weight measures per individual were recorded in Communicare through time and converted within the database to BMI according to the standard formula BMI = weight (kg)/height (m^2^). DNA was prepared from saliva samples collected into Oragene tubes (DNA Genotek, Ontario, Canada) from 405 consenting family members who were available at the time of visits by the study team during the two-year collection period of the study.

### Ethical approvals

Ethical approval for the study was obtained from the Western Australian Aboriginal Health Ethics Committee (WAAHEC; Reference 227 12/12), who reviewed and approved forms for informed consent. Each individual (or the parent or guardian of individuals less than 18 years of age) signed separate informed consent forms to participate in the study and to provide a DNA sample. Following feedback of results to the community, permission to publish was provided by the Board of the local Aboriginal Health Service, which comprised elders representing the extended families residing in the area. Permission to lodge de-indentified genotype and basic demographic data (broad geographical location, age, sex and phenotype information) in the European Genome-phenome Archive (accession number EGAS00001001004) was also obtained from the Board of the local Aboriginal Health Service.

### Genotyping, quality control, and analysis of population structure

DNA from the 405 consenting individuals were genotyped on the Illumina Omni2.5 BeadChip (outsourced to the Centre for Applied Genomics, Toronto, Ontario, Canada). Quality control (QC) data from the service provider indicated a call rate of (mean [SD]) 99.680% [0.005%]. Further in-house QC procedures at the level of individuals resulted in one individual being dropped due to a missing data rate >5%, 2 exclusions due to unintentional duplication, and no exclusions due to outlying heterozygosity (using 3 standard deviation limits), discordant sex, or divergent ancestry (since mixed models were employed to take account of ancestry, cf. below). This provided a post-QC dataset of 402 family members, including 361 with repeated body mass index measurements and 391 (89 cases, 302 family members unaffected at the time of collection) with information on doctor diagnosed T2D as per criteria outlined above. SNPs with minor allele frequency (MAF) <0.05, or with more than 5% missing data, were removed prior to association analysis (cf. below). As family data were employed in our study, complicated by a degree of over-relatedness in the pedigrees, a check of Hardy Weinberg Equilibrium (HWE) was not used as a SNP QC check since it was not clear that we should expect all SNPs to be in HWE. A subset of 70,420 genotyped SNPs with pairwise linkage disequilibrium (LD; *r* ^2^) ≤0.3 and MAF >0.01 was used in principal component analysis (PCA; SMARTPCA within EIGENSOFT [20, 21]) to look at population substructure across the 402 family members.

### Imputation procedures

Pre-phasing of genotyped data was performed using SHAPEIT [22], and imputation conducted using IMPUTE2 [23] with 1000 Genomes (1000G) haplotypes [Phase I integrated variant set release (v3)]. The reference panel includes haplotypes of 1,092 individuals from Africa, Asia, Europe and the Americas. For association analysis, probabilistic imputed SNP genotypes were converted to hard genotype calls provided the posterior probability of the most likely genotype was >0.9, and imputed SNPs were retained only if they had `info’ score >0.5, MAF>0.01 and <10% missing data. To assess imputation accuracy, IMPUTE2 provides measures of concordance and squared Pearson correlation (*r* ^2^) for each genotyped SNP. Directly genotyped SNPs were masked then imputed and concordance between the true genotype and the most likely imputed genotype [where probability prediction threshold > 0.9] for each SNP was calculated [24–26], as well as *r* ^2^ between the true (discrete) allele dosage (coded as 0, 1 and 2 corresponding to the number of minor alleles) and the imputed (continuous) allelic dosages. For low frequency SNPs, *r* ^2^ is the preferred accuracy measure because, unlike concordance, allele frequency is taken into account. We also compared these imputation accuracies for the 195 individuals determined by PCA to represent pure Martu ancestry against the 207 deemed to be of mixed ethnicity by PCA, using the data generated from imputation of the full dataset for the 402 individuals.

### Heritability and association analyses

Heritability for mean BMI was determined from self-reported pedigree structures using the QTDT software package [27], and separately on the basis of kinship and IBD sharing from SNP-chip data calculated using the R package GenABEL v1.7–6 [28] and using the genome-wide complex trait association (GCTA) tool [29]. To provide a visual display of relatedness across the 402 genotyped individuals, the genomic kinship matrix was first calculated in GenABEL v1.7–6 [28], and then converted to a distance matrix followed by hierarchical cluster analysis using single linkage on the dissimilarities. The ape package [30] was used to produce a radial tree plot for the 402 genotyped individuals. For association analyses, the FAmily-based Score Test for Association (FASTA) [31] in GenABELv1.7–6 [28] was employed that uses a linear mixed model (LMM) approximation to model the trait outcome, with whole genome data used to estimate kinship (in order to account for relatedness) and to take account of population substructure. Analyses were carried out using quantitative trait data (BMI), and separately to compare all T2D cases with all unaffected individuals. Association results for genotyped data obtained using GenABEL were compared with results using FaST-LMM [32]. Both GenABEL (FASTA) and FaST-LMM model disease status (control/case for T2D, coded 0/1) as if it were a normally distributed quantitative variable, which has been shown [33] to produce a valid test with respect to testing the null hypothesis of no association. Power calculations using QUANTO [34] show that 361 individuals with BMI measures provide a maximum of 80% power to detect associations at genome wide significance levels (*P*<5×10^-8^), or a maximum of 97% power at suggestive significance levels (*P*<1×10^-5^), for SNPs with MAF>0.3 conferring allelic effects (betas) of magnitude half a unit of standard deviation. For effects (betas) of magnitude 0.25 units of standard deviation, the maximum powers achievable are lowered to 1% and 9% respectively. The 89 T2D cases and 302 unaffected family members provide a maximum of 32% power to detect associations at genome wide significance levels (*P*<5×10^-8^), or 71% power at suggestive significance levels (*P*<1×10^-5^), for SNPs with MAF>0.3 conferring allelic odds ratios >2.5. For odds ratios of 1.5, the powers are lowered to 1.4% and 0.06% respectively.

Manhattan plots were generated using the mhtplot() function of ‘gap’, a genetic analysis package for use in R (see URLs). Quantile-quantile (Q-Q) plots were generated, and inflation factors (denoted λ) calculated in R version 2.15.0 by dividing the median of the observed chi-squared statistics by the median of the theoretical chi-squared distribution. Regional plots of association were created using LocusZoom [35] in which—log_10_ *P*-values were graphed against their chromosomal location. Pairwise LD patterns between all regional SNPs and the top SNP were calculated specifically for this Australian Aboriginal study data using 146 unrelated individuals from the total sample of 402 genotyped individuals. The 146 unrelated individuals were selected by iterative removal of the individuals with the greatest number of estimated relationships with IBD>0.1875.

### Bioinformatic analysis

Global alignment of genomic sequence for the region *SLC28A3* to *NTRK2* from human, cow and mouse was undertaken to locate evolutionarily conserved non-coding sequences (CNS) that might contain regulatory elements and transcription factor binding sites (TFBS). Genomic sequences for the three organisms were exported in FASTA format from ENSEMBL (Genome Reference Consortium Release 37, Ensembl Release 67) and associated annotation exported in the form of a General Feature File (GFF) file. The global alignment tool Multi-LAGAN [36, 37] was used to align genomic sequences using the guide tree (((human) cow) mouse). SYNPLOT [38] was used to visualize the annotated alignment. CNS, defined here as regions with a nucleotide sequence conservation level of ≥0.7 (i.e. ≥ to the least conserved exon sequence in the genes flanking the region of interest), were analysed for promoter and enhancer elements using PROMO [39], AliBaba v2.1 [40], and MatInspector v8.0.5 [41] with a matrix similarity parameter >0.75. We also used the UCSC genome-browser (see URLs) with custom tracks to assess where selected top-hit SNPs are located in relation to elements such as CpG islands or repeat elements (LINES; SINES). To look *de novo* for CpG island-like elements, we firstly masked repeat regions using RepeatMasker (see URLs) following which putative CpG islands were searched for using CpGIsland Searcher [42] with parameters set at 50%GC, 0.60 observed CpG/expected CpG ratio, and length 200bp (rather than the default settings of 55%, 0.65, and 500bp).

## Results

### Characteristics of the study population

The 402 post-QC genotyped individuals used in the GWAS belonged to a small number of inter-related extended pedigrees, as depicted in the radial plot which shows hierarchical clustering of estimated pairwise identity-by-descent allele-sharing (S1 Fig.). Principal component analysis (S2 Fig.) demonstrated a degree of introgression of predominantly Caucasian origin, with a tight cluster of 195 individuals of Martu Aboriginal ancestry across all age groups. Linear mixed models were used in the genetic analysis to take account of both family relationships and this genetic substructure. S1 Table provides basic demographic data (age, sex; at the time of diagnosis and/or collection) for the 391 individuals used in the genetic analyses. De-identified BMI data was also available for 1020 self-reporting Aboriginal individuals in the population-specific Communicare database.

### BMI in the study population

**Fig 1:**
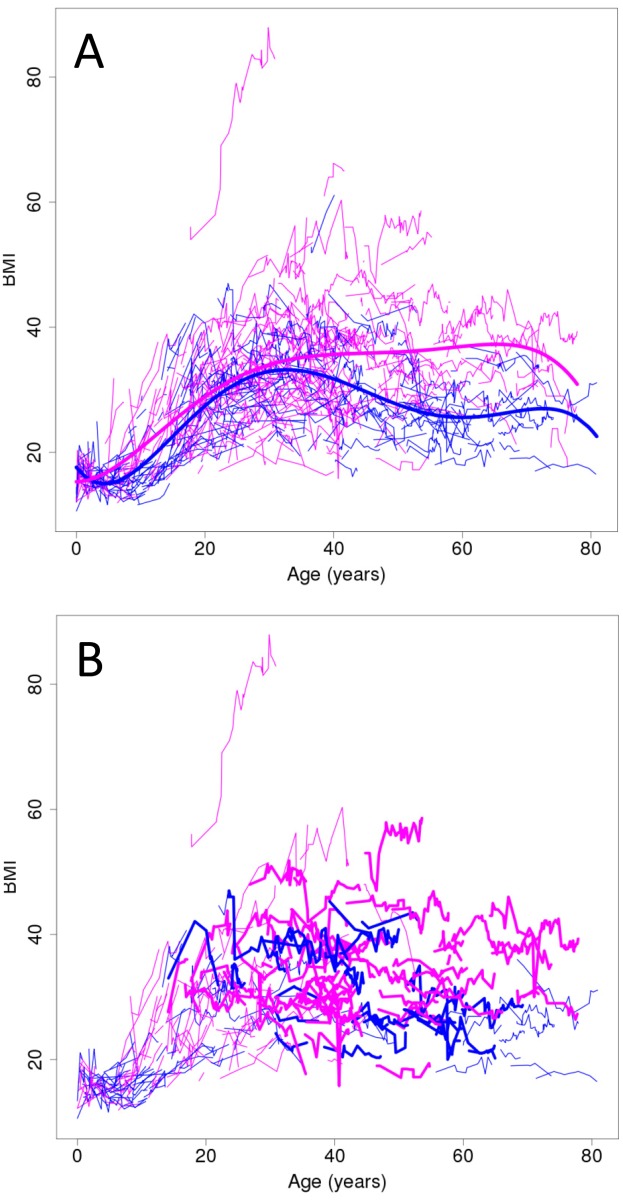
BMI by age plotted using the R package SITAR. (A) plot of all records for self—reporting Aboriginals (N = 1020) in the Aboriginal Health Service’s Communicare database; and (B) plot of all records for the 391 genotyped individuals contributing to association analyses for BMI and T2D. Separate lines trace multiple measurements over time per individual; females (pink) and males (blue). The heavy lines in (A) show the polynomial quintic (power of 5) curves for females (pink heavy line) and males (blue heavy line) that best fit the data. Fitting separate (by gender) curves to the data provided a significantly better fit (*P* = 10^-9^) than fitting a single curve. The extreme outlier was not used in fitting the female polynomial curve. In (B), individuals with T2D are shown in heavy lines.

A BMI >22kg/m^2^ is a significant risk factor for T2D in Australian Aboriginal populations [16]. Fig. 1 part A provides a plot of all BMI measurements for self-reported Aboriginal individuals in the study population (N = 1020). BMI by age was plotted using the R package SITAR [43] for all records in Communicare. Separate lines trace multiple measurements over time per individual; females (pink lines) and males (blue lines). The heavy lines show the polynomial quintic (power of 5) curves for females (pink heavy line) and males (blue heavy line) that best fit the data. Fitting separate (by gender) curves to the data (Fig. 1 part A) provided a significantly better fit (*P* = 10^-9^) than fitting a single curve with a sex-specific displacement. We constructed standardized residuals from these curves by subtracting the observed BMI value from the fitted curve and dividing by the estimated standard deviation (calculated within 1-year age brackets). All GWAS analyses of BMI (cf. below) were carried out using these standardized residuals. The extreme female outlier was not used in fitting the female polynomial curve or in any of the GWAS analyses. This analysis of BMI shows (a) that the majority of the adult population have BMI >22 and are therefore at increased risk of diabetes; (b) a proportion of 15–20 year olds also fall into this category; and (c) there are many individuals in the community who can be classified as overweight (BMI 25.00–29.99) or obese (including class I obesity BMI 30.00–34.99; severe or class II obesity BMI 35–39.99; and morbid or class III obesity BMI≥40) according to Center for Disease Control (CDC) [44] and World Health Organization (WHO) [45] international criteria. By estimating familial correlations based on self-reported pedigree structures using QTDT [27], we estimated heritability for mean BMI in this population to be 55%, broadly in line with other populations internationally [46–48]. Estimation of heritability on the basis of kinship and IBD sharing from the SNP-chip data using the R package GenABEL [28] gave a lower estimate of 38%, consistent with the estimate of 39% (95% confidence interval [17%-61%]) provided by GCTA [29].

### T2D in the study population

Our dataset for the study population (Fig. 1 part B) showed that >75% of adults (≥20 years of age) fall above the BMI cut-off of >22 for increased risk of T2D in Aboriginal Australians [16]. Fig. 1 part B also highlights BMI curves for individuals in the study sample with T2D (heavy pink lines for females; heavy blue lines for males). This demonstrates that our study population is characterized by T2D predominantly associated with high risk BMI measurements, including a number of cases <20 years of age. S2 Fig. part D highlights on a PCA plot the individuals with T2D used in the GWAS. Of the 391 individuals used in the T2D GWAS (cf. below), there were 65 with T2D from 191 individuals (34%) that fell within the pure Martu ancestry cluster (S2 Fig. parts E and G), and 24 with T2D from 200 individuals (12%) of mixed ethnicity. There was insufficient power to analyse GWAS data separately for T2D associated with low to normal (<25; N = 10) BMI or with low (<20 years of age; N = 5) age at onset. There will be some loss of power in the analysis with the inclusion of unaffected individuals less than 20 years of age (since ~10–20% of them may go on to get T2D[16]). This had to be balanced against the gain in power with the increased sample size and greater contribution of individuals in controlling for population substructure and relatedness in the analysis. S1 Table shows the age range and BMI for unaffected individuals <20 and >20 years of age separately. Importantly, there will be no false positive associations generated by including all unaffected individuals in the GWAS analysis for T2D (cf. below).

### GWAS analyses for BMI based on genotyped SNPs

Longitudinal BMI data available for the 361 post-QC individuals with both BMI measurements and genotyped SNP-chip data were representative of the full range of BMI values in the population (Fig. 1 part B). To identify loci associated with BMI we undertook a GWAS using 1,075,436 post quality control genotyped SNPs in these 361 individuals. For longitudinal BMI data (designated BMI-longitudinal), the analyses in GenABEL and FaST-LMM used each individual BMI reading as a separate observation, using the standardized residual from the fitted curve as the trait of interest and modelling the correlation between readings via the estimated kinship. This does not fully account for the correlation between readings as readings from the same person are likely to be more correlated, especially since they are often measured at frequent (close together) time points. The Genomic Control deflation factor [49] was therefore employed in GenABEL to avoid inflation of the overall distribution of test statistics; this correction was not found to be necessary in FaST-LMM, as previously noted [50]. Association analyses were also undertaken in GenABEL and FaST-LMM using the mean of all BMI readings for an individual (designated BMI-mean) as the trait. Although this raises issues of differential variation in BMI-mean due to uneven numbers of readings between individuals, results from these two different analyses were well correlated. The plots presented at S3 Fig. demonstrate that association results for BMI-mean and BMI-longitudinal obtained using FASTA in GenABEL (S3 Fig. part A) or using FaST-LMM (S3 Fig. part B) were highly correlated. Therefore detailed results are given only for BMI-longitudinal (hereinafter referred to as BMI). Similarly, results obtained for each phenotype from GenABEL and FaST-LMM were strongly concordant (S4 Fig.), consistent with our recent evaluation of a range of software implementations for linear mixed models [50]. Q-Q plots (S5 Fig.) for GenABEL (λ BMI-longitudinal = 1.0; λ BMI-mean = 1.02) and FaST-LMM (λ BMI-longitudinal = 1.00; λ BMI-mean = 1.00) analyses also showed equivalent inflation factors. Therefore detailed results presented hereafter are given only for analyses undertaken using FASTA in GenABEL.

**Fig 2:**
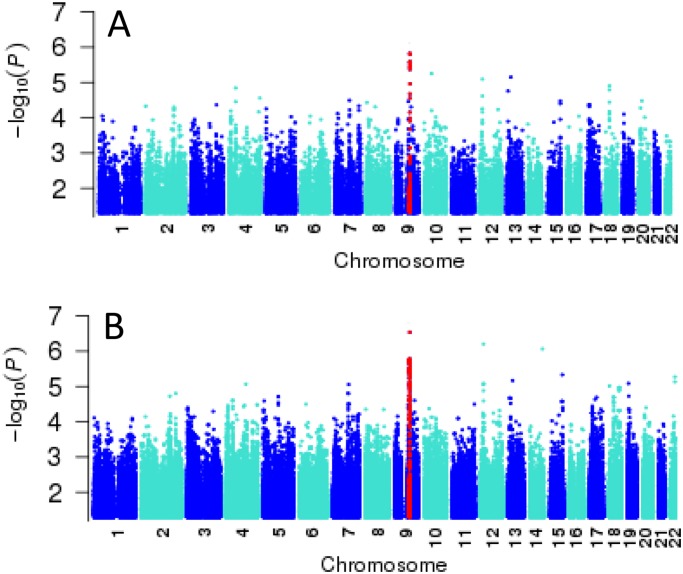
Manhattan plots of genome-wide association results for BMI undertaken using FASTA in GenABEL. (A) results for genotyped data; and (B) results for 1000G imputed data. SNPs in red show the region of the top association in this discovery GWAS.

**Fig 3:**
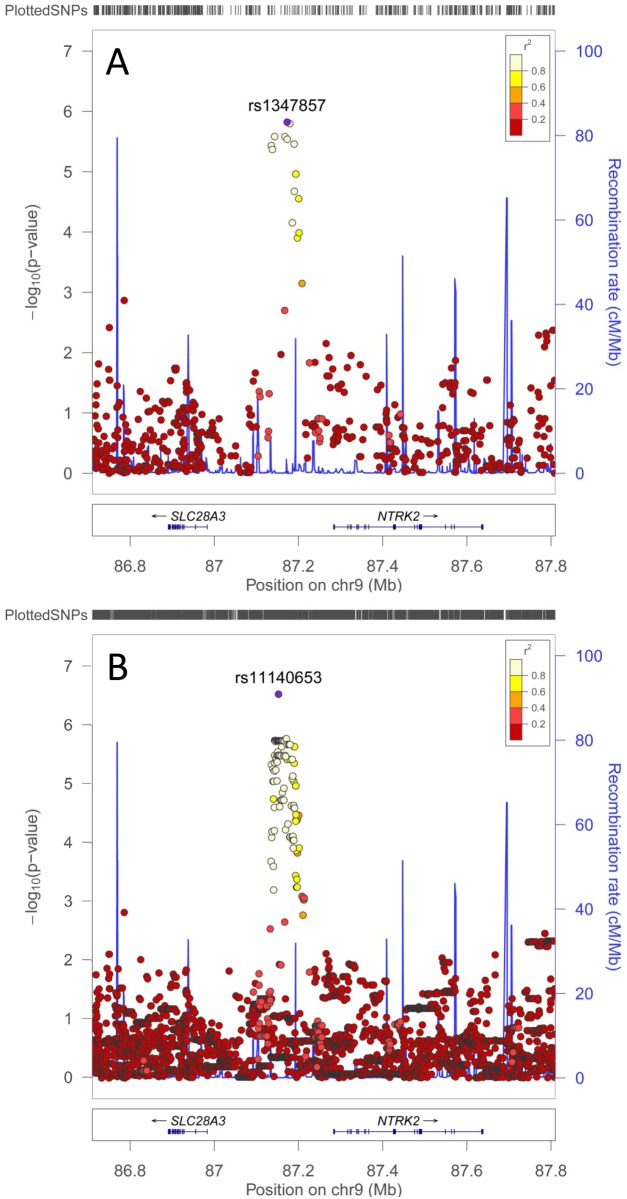
Regional association plots (LocusZoom [35]) of the signal for BMI association in the region *SLC28A3* to *NTRK2* on chromosome 9. (A) is the plot for genotyped data; and (B) is the plot for 1000G imputed data. The −log_10_ *P*-values are shown on the upper part of each plot. SNPs are colored (see key) based on their *r* ^2^ with the labeled hit SNP (purple), calculated in the 146 unrelated genotyped individuals. The bottom section of each plot shows the genes marked as horizontal lines. The second Y axis is for recombination rate, as shown in blue on the plot.

A Manhattan plot showing the genome-wide results for BMI based on the genotyped data is presented in Fig. 2 part A. The top hits did not achieve genome-wide significance, commonly accepted as *P*<5×10^-8^ [51] and concordant with the number of post-QC SNPs (*P* = 0.05/1,075,436 or 4.65×10^-8^) used for this analysis of genotyped data. No specific SNPs reported to achieve genome-wide significance for association with BMI in other populations achieved *P*<10^-2^ in our study, as determined by interrogation of the NIH NHGRI Catalogue of GWAS studies [52]. Nevertheless, since we cannot assume similar patterns of linkage disequilibrium or directionality of associations in our Australian Aboriginal population, we have provided information in S2 Table on SNPs with *P*<10^-2^ in our study population for the genes previously reported to achieve genome-wide significance (i.e. P<5×10^-8^) for association with BMI in other populations. Notably (cf. below), *MC4R* (top SNP rs129959775; *P*_genotyped_ = 4.49×10^-4^) was present in this list. Extending the table to include genes previously reported in GWAS for BMI at *P*<10^-5^ in other populations provided evidence for association at *CNTNAP2* (top SNP rs6960319; *P*_genotyped_ = 4.65×10^-5^). SNPs at *CNTNAP2* were also among the top 50 SNPs from the BMI analysis in our study population (S3 Table). Data for *CNTNAP2* and other top SNPs in genes of potential functional relevance, i.e. in pathways previously identified to be associated with metabolic diseases, are shown in Table 1. Effect sizes (betas 0.35 to 0.72) for these associations are concordant with our power to detect allelic effects of magnitude half a unit of standard deviation of the trait. The top hit (rs10868204; *P*_genotyped_ = 2.73×10^-6^) for BMI in our study population lies in the intergenic region (Fig. 3; cf. below) 5’ of *NTRK2* encoding the type 2 neurotrophic tyrosine kinase receptor for brain-derived neurotrophic factor (BDNF) that regulates energy balance downstream of melanocortin-4 receptor (MC4R). Both *BDNF* and *MC4R* have previously been shown to be associated with obesity in other populations (see S2 Table). Other top hits in our population that are of functional interest (Table 1) occurred in *RBM7* (rs6848632 *P*_genotyped_ = 1.43×10^-5^) which has been related to pancreatic function [53], and at *PIK3C2G* (rs12816270 *P*_genotyped_ = 8.06×10^-6^) which has previously been associated with T2D [54].

**Table 1:** Top GWAS SNP hits in genes of functional relevance for BMI, organized by chromosome.

					BMI Genotyped			BMI Imputed 1000G				
Chromosome	SNP	NCBI37	A1	A2	effB	se_effB	P_deflated_	effB	se_effB	P_deflated_	SNP Location	HGNC*
4	rs6848632	40509521	A	C	0.52	0.08	1.43E-05				INTRONIC	RBM47
	rs6848632	40509521	A	C				0.51	0.08	2.50E-05	INTRONIC	RBM47
7	rs6960319	147258631	G	A	-0.36	0.06	4.65E-05				INTRONIC	CNTNAP2
	rs13225016	147256110	G	A				-0.35	0.06	6.57E-05	INTRONIC	CNTNAP2
**9**	**rs1347857**	**87173097**	**A**	**C**	**0.44**	**0.06**	**1.50E-06**				**INTERGENIC**	**SLC28A3/NTRK2**
	**rs11140653**	**87152509**	**C**	**T**				**0.48**	**0.06**	**2.90E-07**	**INTERGENIC**	**SLC28A3/NTRK2**
12	rs12816270	18770577	A	G	0.72	0.11	8.06E-06				INTRONIC	PIK3C2G
	rs10841048	18779259	T	C				0.71	0.10	6.28E-07	INTRONIC	PIK3C2G

Results are for GWAS analysis in GenABEL using each individual BMI reading as a separate observation, modelling the correlation between readings via the estimated kinship and using the Genomic Control deflation factor to avoid inflation of the overall distribution of test statistics. Results are for allele-wise tests under an additive model of inheritance for genotyped SNPs and for imputed data. Bold indicates the top hit for BMI based on both genotyped and 1000G imputed data. Full lists of the top 50 hits for genotyped SNPs, and the top 100 SNPs for imputed data, appear in S3 Table and S6 Table, respectively.

* Genes separated by forward slash indicate nearest protein coding genes upstream/downstream of the SNP. NCBI37 = bp location on chromosome for NCBI Build 37. A1 = major allele; A2 = minor allele.

### GWAS analyses for T2D based on genotyped SNPs

A Manhattan plot showing the GWAS results for T2D for genotyped SNPs is presented in S6 Fig. part A. Again, the top hits based on analysis of genotyped SNPs did not achieve genome-wide significance for T2D, which was less well-powered than the BMI analysis. In addition, no specific SNPs reported to achieve genome-wide significance for association with T2D in other populations achieved *P*<10^-2^ in our study, as determined by interrogation of the NIH NHGRI Catalogue of GWAS studies [52]. Nevertheless, as for BMI we cannot assume similar patterns of linkage disequilibrium or directionality of associations in our Australian Aboriginal population. Therefore, we have provided information in S4 Table for genotyped SNPs with *P*<10^-2^ in our study population for the genes previously reported to achieve genome-wide significance (i.e. P<5×10^-8^) for association with T2D in other populations. Five genes (*ANK1*, *TSPAN8*, *PROX1*, *GLIS3* and *UBE2E2*) had genotyped SNPs with *P*<10^-3^. Of note, no SNPs at *P*<10^-2^ were observed for *TCF7L2* [55–59] or *KCNJ11* [60], genes that have previously been associated with T2D across multiple ethnicities. However, 4 genes (Table 2) represented in the top 50 SNPs (S5 Table) are supported by strong biological candidacy in related gene pathways. The top hit (rs11240074 *P*_genotyped_ = 5.59×10^-6^) for T2D in our study population lies 5’ of *BCL9* encoding a protein that, along with *TCF7L2*, promotes beta-catenin’s transcriptional activity in the WNT signaling pathway. Additional hits (1.07×10^-4^≤ *P*_genotyped_ ≤4.55×10^-5^) of functional interest occurred in genes involved in pancreatic (*KCNJ6* [61], *KCNA1* [62]) and/or GABA (*GABRR1* [63], *KCNA1* [64]) functions.

**Table 2:** Top GWAS SNP hits in genes of functional interest for T2D, organized by chromosome.

					Genotyped				Imputed 1000G					
Chromosome	SNP	NCBI37	A1	A2	effB	se_effB	chi2.1df	P1df	effB	se_effB	chi2.1df	P1df	SNP Location	HGNC*
**1**	**rs11240074**	**146996480**	**A**	**C**	**0.21**	**0.05**	**20.62**	**5.59E-06**					**INTERGENIC**	**CHD1L/BCL9**
	**rs11240074**	**146996480**	**A**	**C**					**0.21**	**0.05**	**20.58**	**5.73E-06**	**INTERGENIC**	**CHD1L/BCL9**
6	rs6930407	89905239	A	G	0.13	0.03	16.63	4.55E-05					INTRONIC	GABRR1
	rs9451177	89905172	A	G					0.13	0.03	17.37	3.08E-05	INTRONIC	GABRR1
12	rs11063387	4998536	G	C	0.14	0.03	15.50	8.24E-05					INTERGENIC	KCNA6/KCNA1
	rs11063385	4997051	C	T					0.13	0.03	14.97	1.09E-04	INTERGENIC	KCNA6/KCNA1
21	rs8128418	39188732	G	A	0.25	0.06	15.01	1.07E-04					INTRONIC	KCNJ6
	rs113713721	39185153	G	A					0.26	0.06	15.99	6.38E-05	INTRONIC	KCNJ6

Data analyzed in GenABEL using genotyped or 1000G imputed data for all individuals with doctor-diagnosed T2D. Results are for allele-wise tests under an additive model of inheritance. Bold indicates top hit for T2D based on both genotyped and imputed data. Full lists of the top 50 hits for genotyped SNPs, and the top 100 SNPs for imputed data, appear in S5 Table and S7 Table, respectively.

* Genes separated by forward slash indicate nearest protein coding genes upstream/downstream of the SNP. NCBI37 = bp location on chromosome for NCBI Build 37. A1 = major allele; A2 = minor allele.

### GWAS analyses based on imputed SNP data

**Fig 4:**
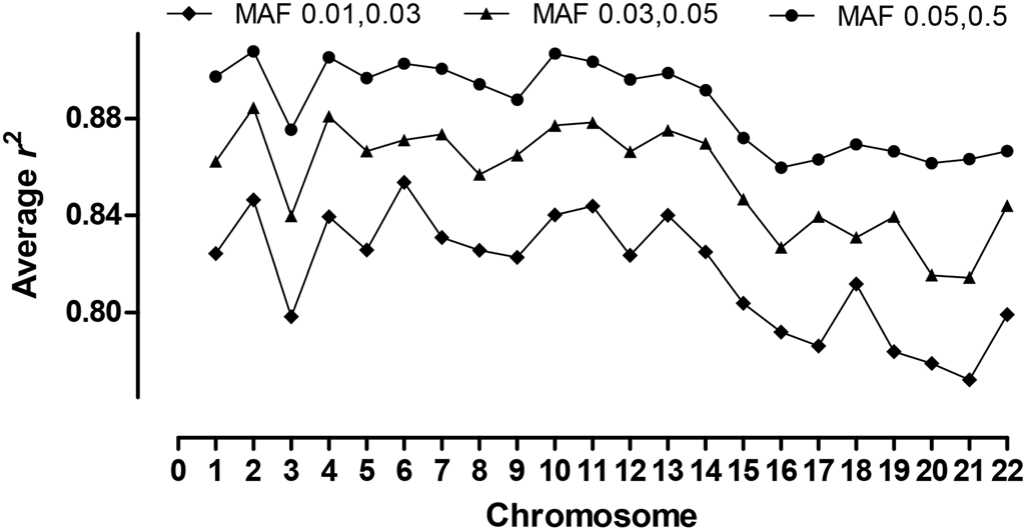
Imputation accuracy for 402 genotyped individuals imputed against the 1000G reference panel. Imputation accuracy is measured as average *r* ^2^ across all autosomes for SNPs of different MAFs (see key).

Recent reports have focused on improving tools for imputation of SNPs based on both the 1000 genomes (1000G) and HapMap project data [23, 65]. It was of particular interest to determine how well 1000G imputation would work for this Australian Aboriginal population, given that most estimates suggest the arrival of Aboriginal people in Australia more than 45,000 years ago [66, 67]. We therefore examined the efficiency of imputing genotypes across the genomes of our study population using 1000G data. High average concordance (92.8%-97.9%) was observed across all chromosomes independently of MAF. Fig. 4 compares the efficiency of imputation across all chromosomes, as measured by imputation *r* ^2^, for SNPs at different MAF. As expected, *r* ^2^ is lower for SNPs with MAF 0.01–0.03 (mean *r* ^2^ 77.2–85.4) compared to SNPs with MAF 0.03–0.05 (mean *r* ^2^ 81.5–88.5%) and MAF 0.05–0.5 (mean *r* ^2^ 85.9–90.7%). When examined across individuals, mean imputation accuracy for the 195 individuals of pure Martu ancestry (concordance 94.8; *r* ^2^ 88.8) were not different to measures for the 207 individuals of mixed ethnicity (concordance 95.6; *r* ^2^ 90.0) or to measures across all 402 individuals (concordance 95.2; *r* ^2^ 89.5).

In relation to the GWAS analyses for BMI (Fig. 2 part B; Fig. 3 part B; Table 1; S6 Table), 1000G imputation improved significance for associations at *NTRK2* (Fig. 3 part B; Table 1: *P*_genotyped_ = 1.50×10^-6^; *P*_imputed_1000G_ = 2.90×10^-7^) and at *PIK3C2G* (Table 1: *P*_genotyped_ = 8.06×10^-6^; *P*_imputed_1000G_ = 6.28×10^-7^) but not at other genes of functional interest (Table 1). Similarly, for the T2D imputed GWAS analysis (S6 Fig. part B; S7 Table), top SNPs for 1000G imputed data in genes of functional interest (Table 2) were generally of the same order of magnitude as top genotyped SNPs, including support for the top hit at *BCL9*. Novel findings in the top 100 SNPs for 1000G imputed data included a hit at the previously identified gene for T2D *IGF2BP2* (S4 Table: rs138306797, *P*_imputed_1000G_ = 2.55×10^-6^) that had not achieved *P*<10^-2^ in the genotyped data, and hits at *P*_imputed_1000G_ <10^-6^ in genes not previously associated genetically or functionally with T2D (S7 Table: *SATB2/TYW5* on chromosome 2, *MTHFD1L/AKAP12* on chromosome 6; *KLF5/KLF12* on chromosome 13).

### Genes for obesity or for T2D?

Analysis of T2D and the standardized BMI residual (BMI-longitudinal) was initially performed without any covariate adjustment. Since T2D and BMI are highly correlated and have been shown to have shared genetic influences [68], the question arises as to which phenotype is primarily regulated by the genes of interest identified in this study. We therefore repeated the analyses allowing for BMI and T2D as covariates (S1 Text). Results demonstrated that (A)—log_10_ *P*-values for T2D adjusted for the standardized BMI residual were highly correlated with the original unadjusted analysis for both genotyped and imputed data, and (B) conversely,—log_10_ *P*-values for unadjusted BMI and BMI adjusted for T2D were likewise highly correlated. This suggests that genetic effects on T2D as originally calculated are largely independent of any effects attributable to the mean standardized BMI residual. Specifically, the significance levels for genes of functional interest are of the same order of magnitude in both the adjusted (S1 Text) and the original analysis (Table 2). Similarly, genetic effects on BMI as originally calculated are largely independent of any effects attributable to T2D, consistent with the regional association plots (Fig. 3, S7 Fig.) where no evidence for association is seen in plots comparing T2D results across regions that contained the top hits for BMI. This includes the regions containing *RMB7*, which previously has been related to pancreatic function [53], and *PIK3C2G* which has been associated with T2D in another population [54].

### Interrogating the *SLC28A3* to *NTRK2* intergenic region

**Fig 5:**
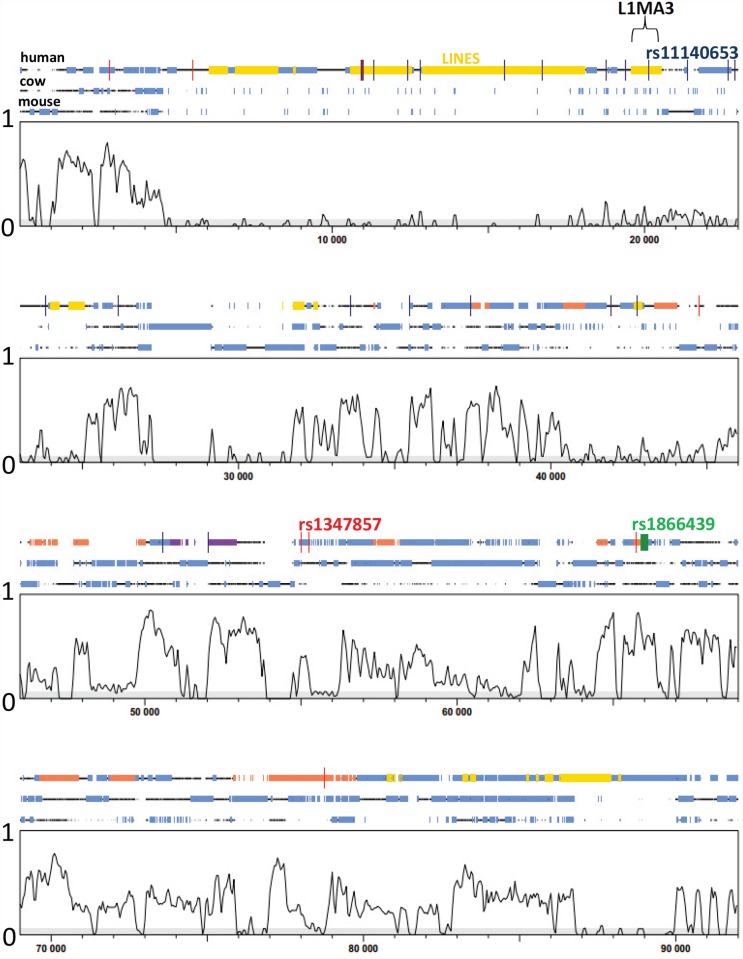
SYNPLOT [38] for a section of the intergenic region (NCBI Build 37: 87,140,000bp to 87,190,000bp) ~94kb upstream of *NTRK2* on chromosome 9q21.33. Since regulatory elements are usually found with conserved regions of the genome, we interrogated the region of our top hits using this *in silico* analysis to look for conserved regions across multiple species. The multiple alignments of (top to bottom) human, cow and mouse sequences were performed across the complete intergenic region *SLC28A3* to *NTRK2* using LAGAN. The segment of the alignments shown here is the region that contains the top association hits (*P*<5×10^-6^). The central plotted curves show the degree of conservation of sequence across all three species, on a vertical scale 0–1 (= 100%), such that the peaks represent CNS. CNS are defined here as regions with a nucleotide sequence conservation level of ≥0.7, i.e. ≥ to the least conserved exon sequence in the two genes (not shown on this plot) *SLC28A3* and *NTRK2* flanking the intergenic region of interest. Blue boxes indicate repetitive sequence in all 3 species. The human sequence is also annotated with positions of: (i) LINE-1 elements (yellow); (ii) the top genotyped SNPs (red vertical bars) including the top SNP rs1086204, and the SNP of interest rs1866439; (iii) the top imputed SNPs (blue vertical bars) including the top SNP rs1140653; (iv) the CpG island-like element identified using CpG Island Seacher (green); and (v) positions of peaks of mono-methylation of lysine 4 of histone H3 (H3K4Me1) as measured in NHEK or NHLF cell lines (mauve) or in H1-hESC human embryonic stem cells (orange) by the ENCODE project, as identified using the UCSC browser (see S9 Fig.).

As with most GWAS for common complex diseases [69], top SNP hits identified here were generally located within introns or in potential regulatory regions upstream or downstream of the gene of interest. In the case of the top hit for BMI (Fig. 3), the peak of association was clearly located upstream of the best functional candidate for BMI, the *NTRK2* gene, with little evidence for linkage disequilibrium between SNPs in this peak of association and SNPs within the coding region of the gene. Conditioning on the top genotyped and top imputed SNPs (S8 Fig.) reduced all other SNP signals to *P*>10^-3^, suggesting a single major signal regulating BMI across this region. We therefore interrogated the region of top hits upstream of *NTRK2* to see if we could find evidence for potential regulatory elements. Initially we used SYNPLOT to plot the location of the top genotyped SNPs (*P*<5×10^-6^) in relation to CNS upstream of *NTRK2* (Fig. 5). These 8 genotyped SNPs were all in strong LD with the top SNP (r^2^>0.8; Fig. 3). SNP rs1866439 (*P*_genotyped_ = 1.59×10^-6^) was of equivalent significance to the top SNP rs1347857 (*P*_genotyped_ = 1.5×10^-6^) and was unique in being the only top SNP located within a CNS peak, with the risk allele causing loss of a NHP1 binding site falling within a half-palindromic estrogen response element (TGAGTagtTG^A^/_G_CC) [70, 71]. NTRK2 expression has been shown to be regulated by estradiol [72]. Other indications that this might be a regulatory region, as annotated on Fig. 5, included: (i) the presence adjacent to rs1866439 of a CpG-island (50%GC; observed CpG/expected CpG 61%; 212bp in length); (ii) a number of peaks of mono-methylation of lysine 4 of histone H3 (H3K4Me1; frequently found near regulatory regions) as measured by the ENCODE project (see URLs), including around rs1866439; and (iii) the peak of association with the top imputed SNPs (Fig. 3 part B) falling within a non-conserved region (no CNSs) of fragments of long (LINE-1 or L1) interspersed elements or retrotransposons (Fig. 5 and S9 Fig.). S9 Fig. shows the positions of these top SNPs in relation to the L1 elements, with the top imputed SNP (rs11140653) specifically falling within a L1MA3 element (as also annotated on Fig. 5). While full-length L1s are required for retrotransposition [73], regulatory elements that overlap with fragments of L1 sequence have been shown to be involved in gene silencing [74]. Further functional studies will be required to determine which variants and elements are important in regulating NTRK2 in this Aboriginal population.

## Discussion

Results of the discovery GWAS undertaken here provide the first hypothesis-free insights into genetic risk factors for high BMI and T2D in an Australian Aboriginal population. Although estimates suggest the arrival of Aboriginal people in Australia more than 45,000 years ago [66, 67], we found that we were able to (A) genotype with high accuracy using the Illumina 2.5M Duo SNP chip, and (B) impute genotypes with high accuracy based on the reference panel from the 1000 Genomes project [75]. This included imputation accuracy in both the subset of 195 individuals determined by PCA to be of pure Martu ancestry and in the 207 individuals in whom we observed varying degrees of introgression with Caucasian genomes. This imputation accuracy compares favorably with that reported for imputation based on the 1000G reference panel of African and American ancestry groups genotyped on the Illumina Omni2.5M Duo chips [76]. The major limitation in the present study was sample size and power, but as a pioneering study in the area of application of modern genomics to Aboriginal health in Australia it is important that results of this study are reported in order to allay concerns about genetic research as applied to health outcomes, to boost confidence in the approach, and stimulate replication studies in other Australian Aboriginal populations. The important practical implication of our demonstration that 1000G imputation could be employed with accuracy in this Australian Aboriginal population is that further cost-effective genome-wide approaches can be applied to understanding pathogenesis of complex diseases in Australian Aboriginal populations.

In our study, modest support (P<10^-2^) was obtained for a number of genes previously shown to achieve genome wide significance in other populations (see Tables S2 and S4), but these were generally not represented amongst the top hits observed for our study population. Of note, no SNPs at *P*<10^-2^ were observed for *TCF7L2* [55–59] or *KCNJ11* [60], genes that have previously been associated with T2D across multiple ethnicities (though not with Pima Indians for *TCF7L2* [77]). Recent large-scale trans-ancestry meta-analysis shows a significant excess in directional consistency of T2D risk alleles across ancestry groups [78]. Whilst the power of our study may have limited our ability to replicate top hits found in large scale population-based case-control GWAS and meta-analyses for BMI and T2D, there is increasing support [79, 80] for the possibility that functional variants that are rare in the general population may be enriched through highly shared ancestry, identity-by-descent, and linkage in the kind of extended pedigrees that we have used in our discovery GWAS. Accordingly, our findings here, while requiring further definitive replication in additional Australian Aboriginal populations, highlight interesting novel association signals for BMI and T2D that might provide important clues to disease pathogenesis in this population.

For T2D, the top association in our study population, supported by both genotyped and imputed data, was in the region of *BCL9*. *BCL9* encodes a protein that, along with *TCF7L2*, promotes beta-catenin’s transcriptional activity in the WNT-signaling pathway [81]. WNT-signaling is important in the transcription of proglucagon, which serves as precursor for the incretin hormone glucagon-like peptide 1 that stimulates insulin secretion [82]. Additional hits of functional interest occurred in *KCNJ6*, *KCNA1*, and *GABRR1*. *KCNJ6* lies adjacent to *KCNJ15* on chromosome 21q22.13. KCNJ6 is an ATP-sensitive potassium channel that regulates insulin secretion in pancreatic beta cells in response to nutrients [61]. KCNJ15 is another member of this family of inwardly-rectifying ATP-sensitive potassium channels that has been shown to be associated with T2D in an Asian population [83]. KCNA1 is the voltage-gated potassium channel Kv1.1 that is expressed in pancreatic β-cells and can influence glucose-stimulated insulin release [62]. It has also been associated with ataxia and epilepsy in humans carrying rare variants [84], and mutant mice provide a model for ataxia in which Kv1.1 influences gamma-aminobutyric acid (GABA) release in Purkinje cells of the brain [64]. *GABRR1* is a member of the rho subunit family of GABA A receptors. Previous studies in China have demonstrated association between polymorphisms at *GABRR1* and diabetic cataract [63]. Recently we also demonstrated that polymorphisms in both GABA-A and GABA-B receptor pathway genes are associated with T2D in an extended family from the United Arab Emirates [85]. GABA-A receptors act as inhibitory neurotransmitters in the central nervous system. They are also present in the endocrine part of the pancreas at concentrations comparable to those in the central nervous system, and co-localize with insulin in pancreatic beta cells [86]. Recent work has shown a role for both GABA-A and GABA-B receptors in regulating insulin secretion and glucagon release in pancreatic islet cells from normo-glycaemic and T2D individuals [87].

Amongst our top hits for BMI was *PIK3C2G*, a gene previously associated with T2D and with serum insulin levels in Japan [54]. PIK3C2G is a member of a conserved family of intracellular phosphoinositide 3-kinases known to be involved in a large array of cellular functions. Daimon and coworkers [54] related their association to prior observations [88] of expression of PIK3C2G in the pancreas. However, recent GWAS have observed associations between polymorphisms at *PIK3C2G* and hyperlipidemia and myocardial infarction [89].

A second hit for BMI in our population was *CNTNAP2* that encodes contactin associated protein-like 2 which localizes to the juxtaparanodal region of the nodes of Ranvier in myelinated axons, where it is required for proper localization of the potassium voltage-gated channel KCNA1 [90]. Mutations in *Cntnap2* in mice impair Kv1.1 localization, and can be obesity-promoting or obesity-resistant in diet-induced obesity depending on genetic background [91]. Polymorphism at *CNTNAP2* was among the strongest gene x environment interactions among African-Americans with BMI as outcome, specifically interacting with dietary energy intake [92]. *CNTNAP2* is amongst genes that lie within regions of *de novo* duplications and deletions recently linked to syndromic obesity in children [93].

Perhaps the most convincing association observed in our study was between BMI and SNPs that lie in the intergenic region between *SLC28A3* and *NTRK2* on chromosome 9q21.33. SLC28A3 is a concentrative nucleoside transporter with broad specificity for pyrimidine and purine nucleosides [94], and is therefore not a strong candidate genetic risk factor for BMI. On the other hand, NTRK2 (also known as *TRKB* or tyrosine kinase receptor B) is the receptor for BDNF and regulates energy balance downstream of MC4R. Associations between BMI and SNPs at both *MC4R* [95–97] and *BDNF* [98, 99] have achieved genome-wide significance in multiple independent studies, but association between *NTRK2* and BMI has only previously been observed (P = 1.04×10^-6^) in a gene-centric multi-ethnic meta-analysis of 108,912 individuals genotyped on the ITMAT-Broad-Candidate Gene Association Resource (CARe) array containing 49,320 SNPs across 2100 metabolic and cardiovascular-related loci [100]. Mouse *Bdnf* mutants that express decreased amounts of Ntrk2 show hyperphagia and maturity-onset obesity [101], while risk variants in human *BDNF* were significantly associated with more food servings in a study of obesity susceptibility loci and dietary intake [102]. Deficiency in MC4R signaling reduces expression of BDNF in ventromedial hypothalamic nuclei, indicating that BDNF and its receptor NTRK2 are downstream components in the MC4R-mediated control of energy balance. Overall, our data are consistent with meta-analysis [96] of large-scale GWAS of BMI that, along with data from rare monogenic forms of obesity [103], highlight a neuronal influence on body weight regulation.

In summary—we report here data for the first GWAS of complex disease in an Australian Aboriginal population. Whilst the top hits for BMI and T2D are in novel genes not yet reported for other GWAS, they occur in genes that belong to key pathways strongly supported by previous GWAS in other ethnicities. This means that current international efforts [82, 104, 105] to target these pathways will be relevant and translatable to the Australian Aboriginal population, and the genes identified here may indeed provide novel targets in this endeavour.

## Online Resources

The URLs for web resources presented herein are as follows:

R version 2.15.0, http://www.R-project.org/

The mhtplot() function of ‘gap’ for R, http://www.mrc-epid.cam.ac.uk/Personal/jinghua.zhao/r-progs.htm

UCSC genome-browser, http://ucscbrowser.genenetwork.org/cgi-bin/hgGateway

The Encode Project, http://www.genome.gov/encode/

Smit AFA, Hubley R, Green P (1996–2010) RepeatMasker Open-3.0. http://www.repeatmasker.org

## Supporting Information

S1 Fig Radial plot showing hierarchical clustering of estimated pairwise identity-by-descent allele-sharing for the 402 genotyped individuals used in this study. The genomic kinship matrix was first calculated in GenABEL v1.7–6, and converted to a distance matrix and hierarchical cluster analysis using single linkage on the dissimilarities. The ape package was used to produce radial tree plot.
(PDF)

S2 Fig Principal component (PC) analysis (PCA) plots showing population substructure in the study population. A subset of 70,420 genotyped SNPs with pairwise linkage disequilibrium (LD; *r*2) ≤0.3 and MAF >0.01 was used in PCA (SMARTPCA within EIGENSOFT) to look at population substructure across the 402 genotyped family members. Plots (A) PC1 x PC2, (B) PC1xPC3, and (C) PC2xPC3 show individuals, color coded by age (see key). (D) shows the PC1 x PC2 plot in which individuals are color coded (see key) according to their T2D status. PCA plots (E) PC1 x PC2, (F) PC1xPC3, and (G) PC2xPC3 show individuals color coded by ancestry (see key). Note that the Martu clusters comprise 73 individuals aged < 20, 25 individuals aged 20–30, 57 individuals aged 30–50, and 40 individuals aged > 50. Hence there is no evidence for disproportionate representation in any age class. Reference HapMap populations are not included at the specific request of the Board of the Aboriginal Health Service.
(PDF)

S3 Fig Demonstrates the strongly correlated GWAS results (genotyped data) for mean BMI compared with BMI longitudinal for (A) FASTA GenABEL, and (B) Fast-LMM analyses. (PDF)

S4 Fig Demonstrates the strongly correlated GWAS results (genotyped data) for FASTA GenABEL versus Fast-LMM for (A) mean BMI, and (B) BMI longitudinal. (PDF)

S5 Fig Provides Q-Q plots and associated lambda values for GWAS analyses undertaken for (A) FASTA GenABEL BMI longitudinal, (B) FASTA GenABEL mean BMI, (C) Fast-LMM BMI longitudinal, (D) Fast-LMM mean BMI. (PDF)

S6 Fig Manhattan plots of genome-wide association results for T2D undertaken using FASTA in GenABEL. (A) results for genotyped data; and (B) results for 1000G imputed data. SNPs in red show the region of the top association in this discovery GWAS.
(PDF)

S7 Fig Regional association plots (Locuszoom) of the imputed SNP signals for BMI (upper graph) and T2D (lower graph) in specific regions. (A) *SLC28A3* to *NTRK2* on Chromosome 9; (B) *CNTNAP2* on Chromosome 7; (C) *RBM7* on Chromosome 4; and (D) *PIK3C2G* on Chromosome 12. In each plot the −log10 *P*-values are shown on the upper section, with SNPs colored (see key) based on their *r2* with the labeled top hit SNP (purple), calculated in the 146 unrelated genotyped individuals. Red arrows highlight the position of the BMI hit on the T2D plot. The bottom section of each plot shows the genes marked as horizontal lines.
(PDF)

S8 Fig Regional association plots (Locuszoom) of the signals for (A) BMI genotyped and (B) BMI imputed in the region *SLC28A3* to *NTRK2* on Chromosome 9 before (upper graph) and after (lower graph) conditioning on the top SNPs rs1347857 and rs11140653, respectively. Analysis undertaken using an additive model in FaST-LMM.
(PDF)

S9 Fig Plot generated in the UCSC genome-browser for a section of the intergenic region (NCBI Build 37: 87,140,000bp to 87,190,000bp) ~94kb upstream of *NTRK2* on Chromosome 9q21.33. The plot shows positions of the top (*P*<5×10^-6^) genotyped (in red vertical lines) and 1000G imputed (in blue vertical lines) SNPs, with the top imputed SNP (rs1140653), the top genotyped SNP (rs1347857) and the SNP of functional interest discussed in the main text (rs1866439) shown again on separate rows. These SNPs are shown relative to: (i) the CpG island-like motif identified using CpG Island Searcher; (ii) the various fragments of LINE-1 (L1) elements (with the L1MA3 element in which the top imputed SNP is located shown in blue); and (iii) peaks of histone methylation (H3K4Me1, H3K4Me3) or acetylation (H3K27Ac) measured in 7 cell lines from the ENCODE project.
(PDF)

S1 Table Characteristics of the family-based Aboriginal study sample. The 391 post-QC genotyped individuals used in the GWAS belonged to a small number of interrelated extended pedigrees, as depicted in the radial plot of pairwise identity-by-descent allele-sharing presented in S1 Fig.
Characteristics of the 391 genotyped individuals used in the GWAS are provided here.
(PDF)

S2 Table SNP associations at *P* <0.01 observed in the WA Aboriginal study population for genes previously reported* to achieve *P* <5×10^-8^ (or *P* <10^-5^ shaded grey) for association with BMI in other populations. Bold indicates a SNP hits observed in the imputed but not the genotyped data, including at *DICER* (*P* <10^-6^).
(PDF)

S3 Table Top GWAS autosomal SNP hits for BMI, organised by chromosome. Results for the top 50 hits for “BMI longitudinal” GWAS analysis in GenABEL using each individual BMI reading as a separate observation, modelling the correlation between readings via the estimated kinship and using the genomic control deflation factor to avoid inflation of the overall distribution of test statistics. Results are for allele-wise tests under an additive model of inheritance. Bold indicates SNP associations of functional interest presented in main Table 1.
(PDF)

S4 Table SNP associations observed at nominal *P*<0.01 in the WA Aboriginal study population for genes previously reported to achieve *P* <5×10^-8^ for association with T2D in other populations. Bold indicates SNP hits observed in the imputed but not the genotyped data, including at *IGF2BP2* (*P* <10^-5^).
(PDF)

S5 Table Top 50 GWAS SNP hits for T2D, organised by chromosome. Results are for allele-wise tests under an additive model of inheritance. Bold indicates SNP associations of functional interest presented in main Table 2.
(PDF)

S6 Table Top GWAS imputed SNP hits for BMI, organised by chromosome. Results for the top 100 hits for “BMI longitudinal” GWAS analysis in GenABEL using each individual BMI reading as a separate observation, modelling the correlation between readings via the estimated kinship and using the genomic control deflation factor to avoid inflation of the overall distribution of test statistics. Results are for allele-wise tests under an additive model of inheritance. Bold indicates 3 top SNP associations for imputed data (*P* <10^-6^) coincide with two top genes of functional interest (*NTRK2*, *PIK3C2G*) presented in main Table 1, as well as a hit near *DICER* a previously observed GWAS hit for BMI (see S2 Table).
(PDF)

S7 Table Top GWAS imputed SNP hits for T2D, organised by chromosome. Results are for the top 100 hits for T2D GWAS analysis in GenABEL for allele-wise tests under an additive model. Bold indicates 3 top SNP associations for imputed data (*P*<10^-6^) not observed in the genotyped data. Bold plus grey shading indicates hits that coincide with top gene of functional interest (*BCL9*) presented in main Table 2, as well as a hit near *IGF2BP2* a previously observed GWAS hit for T2D (see S4 Table).
(PDF)

S1 Text Supplement on Conditional Analyses. (PDF)
