# Supplementary material for: First Genome-Wide Association Study in an Australian Aboriginal Population Provides Insights into Genetic Risk Factors for Body Mass Index and Type 2 Diabetes
Source: PLoS One. 2015 Mar 11;10(3):e0119333. doi: 10.1371/journal.pone.0119333 (PMC4356593; doi:10.1371/journal.pone.0119333)

Figure S1. Radial plot showing hierarchical clustering of estimated pairwise identity-by-descent allele-sharing for the 402 genotyped individuals used in this study. The genomic kinship matrix was first calculated in GenABEL v1.7-6, and converted to a distance matrix and hierarchical cluster analysis using single linkage on the dissimilarities. The ape package was used to produce radial tree plot.

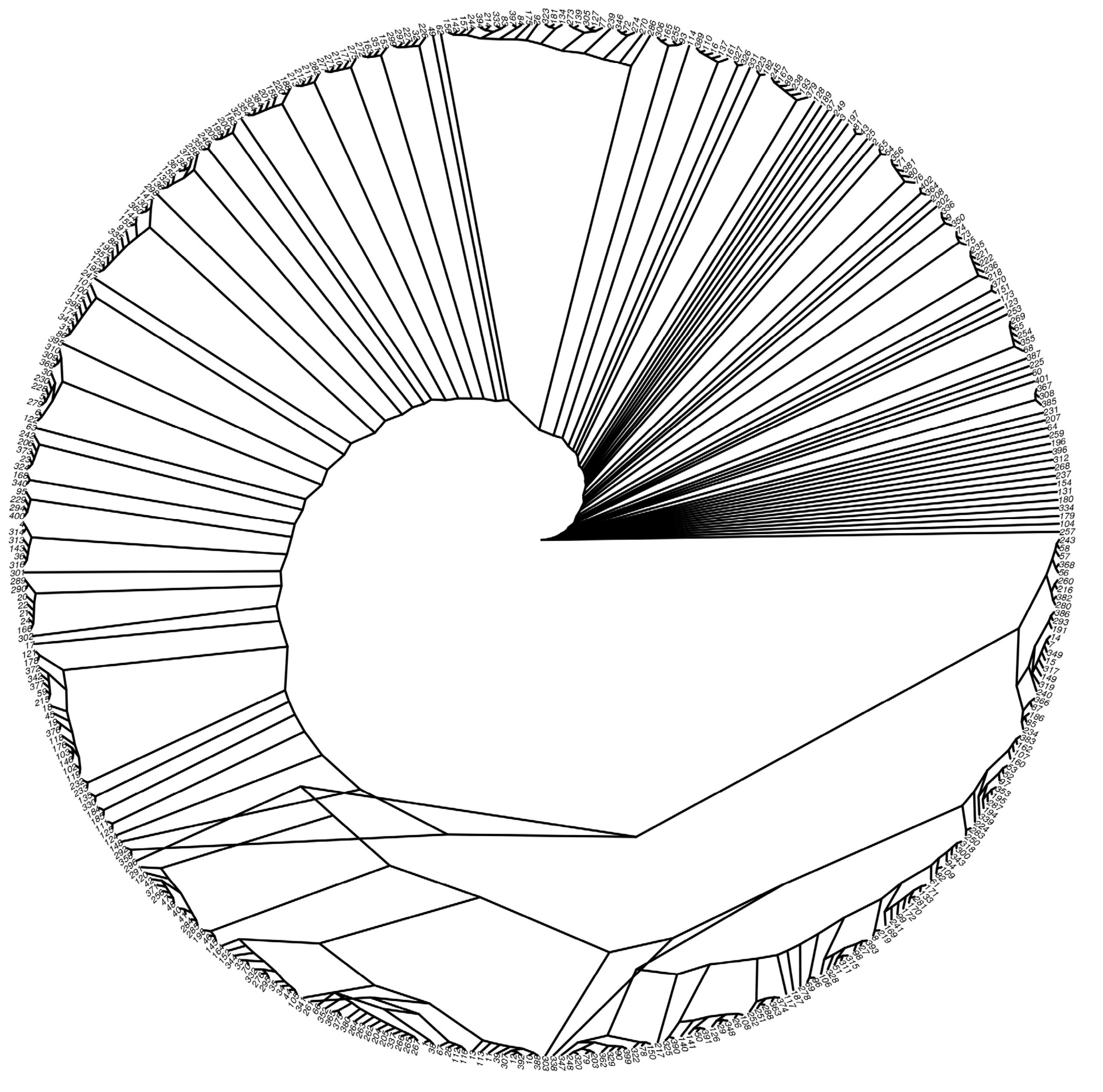

Supplement: S1 Fig — The genomic kinship matrix was first calculated in GenABEL v1.7–6, and converted to a distance matrix and hierarchical cluster analysis using single linkage on the dissimilarities. The ape package was used to produce radial tree plot. (PDF) [file pone.0119333.s001.pdf]
