# Supplementary material for: First Genome-Wide Association Study in an Australian Aboriginal Population Provides Insights into Genetic Risk Factors for Body Mass Index and Type 2 Diabetes
Source: PLoS One. 2015 Mar 11;10(3):e0119333. doi: 10.1371/journal.pone.0119333 (PMC4356593; doi:10.1371/journal.pone.0119333)

Figure S2. Principal component (PC) analysis (PCA) plots showing population substructure in the study population. A subset of 70,420 genotyped SNPs with pairwise linkage disequilibrium (LD; r^2) ≤0.3 and MAF >0.01 was used in PCA (SMARTPCA within EIGENSOFT) to look at population substructure across the 402 genotyped family members. Plots (A) PC1 x PC2, (B) PC1xPC3, and (C) PC2xPC3 show individuals, color coded by age (see key). (D) shows the PC1 x PC2 plot in which individuals are color coded (see key) according to their T2D status.

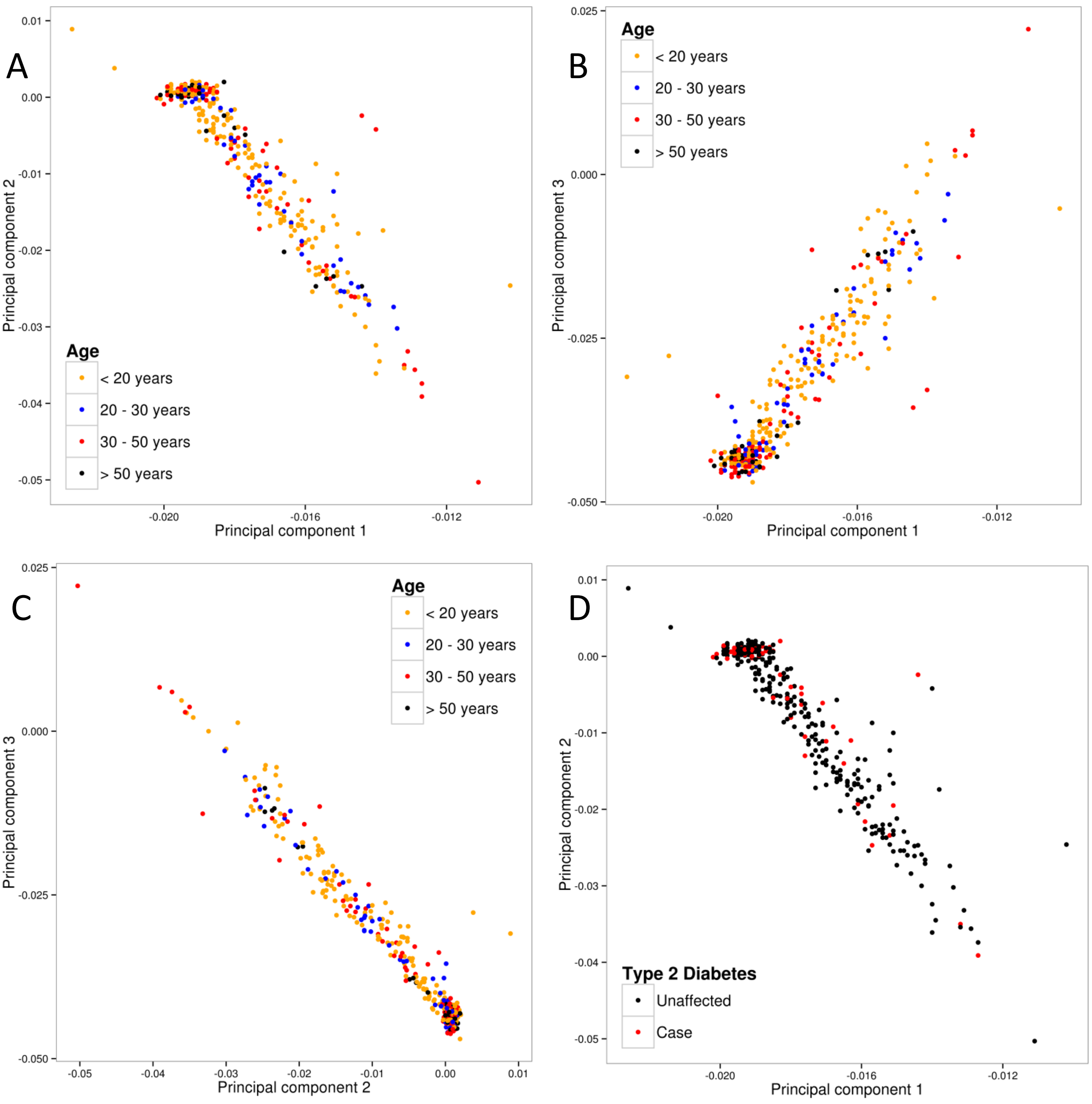

…/cont

Figure S2 continued. PCA plots (E) PC1 x PC2, (F) PC1xPC3, and (G) PC2xPC3 show individuals color coded by ancestry (see key). Note that the Martu clusters comprise 73 individuals aged < 20, 25 individuals aged 20-30, 57 individuals aged 30-50, and 40 individuals aged > 50. Hence there is no evidence for dis-proportionate representation in any age class. Reference HapMap populations are not included at the specific request of the Board of the Aboriginal Health Service.

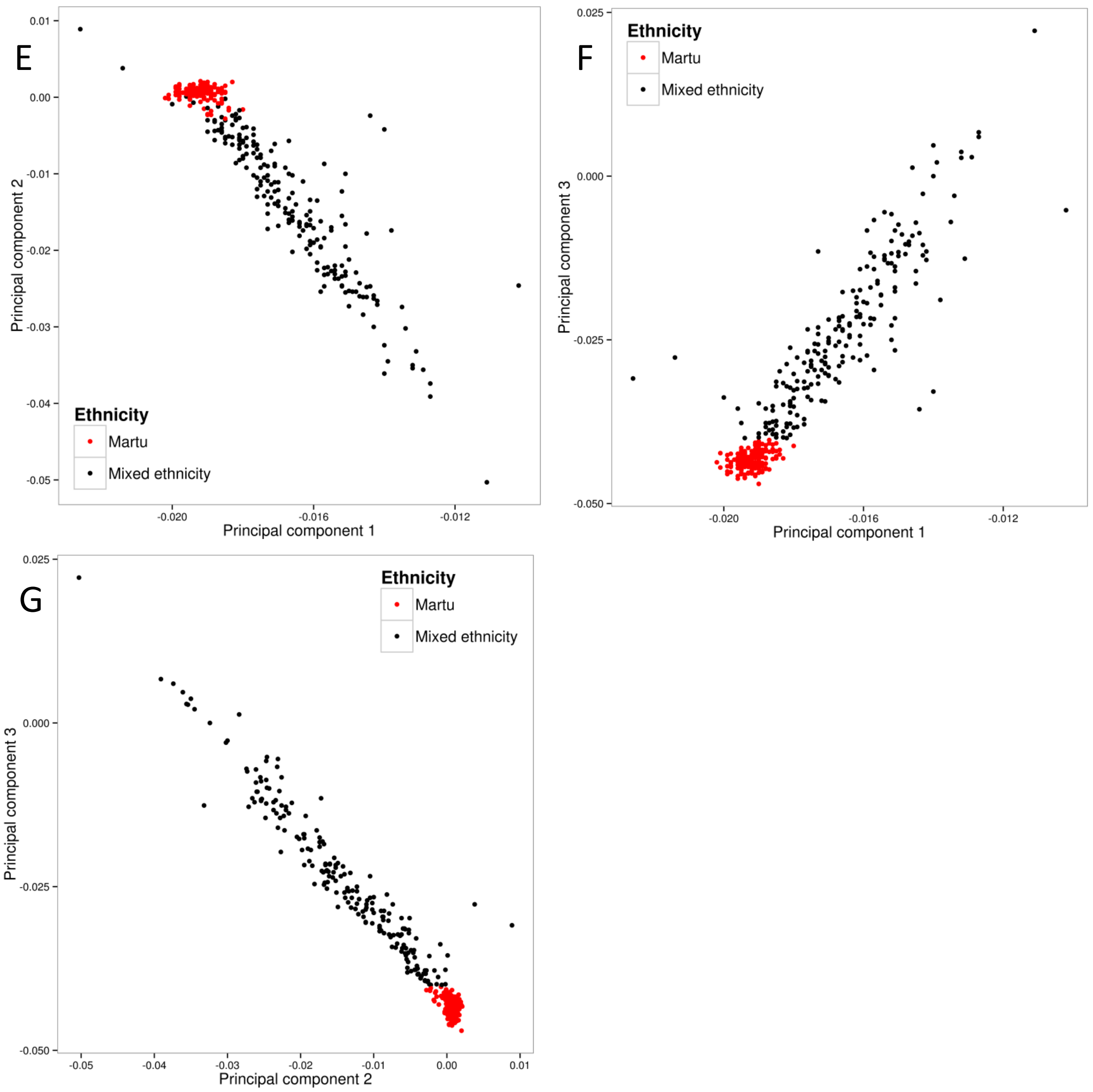

Supplement: S2 Fig — A subset of 70,420 genotyped SNPs with pairwise linkage disequilibrium (LD; r2) ≤0.3 and MAF >0.01 was used in PCA (SMARTPCA within EIGENSOFT) to look at population substructure across the 402 genotyped family members. Plots (A) PC1 x PC2, (B) PC1xPC3, and (C) PC2xPC3 show individuals, color coded by age (see key). (D) shows the PC1 x PC2 plot in which individuals are color coded (see key) according to their T2D status. PCA plots (E) PC1 x PC2, (F) PC1xPC3, and (G) PC2xPC3 show individuals color coded by ancestry (see key). Note that the Martu clusters comprise 73 individuals aged < 20, 25 individuals aged 20–30, 57 individuals aged 30–50, and 40 individuals aged > 50. Hence there is no evidence for disproportionate representation in any age class. Reference HapMap populations are not included at the specific request of the Board of the Aboriginal Health Service. (PDF) [file pone.0119333.s002.pdf]
