# Supplementary material for: First Genome-Wide Association Study in an Australian Aboriginal Population Provides Insights into Genetic Risk Factors for Body Mass Index and Type 2 Diabetes
Source: PLoS One. 2015 Mar 11;10(3):e0119333. doi: 10.1371/journal.pone.0119333 (PMC4356593; doi:10.1371/journal.pone.0119333)

Figure S3. Demonstrates the strongly correlated GWAS results (genotyped data) for mean BMI compared with BMI longitudinal for (A) FASTA GenABEL, and (B) Fast-LMM analyses.

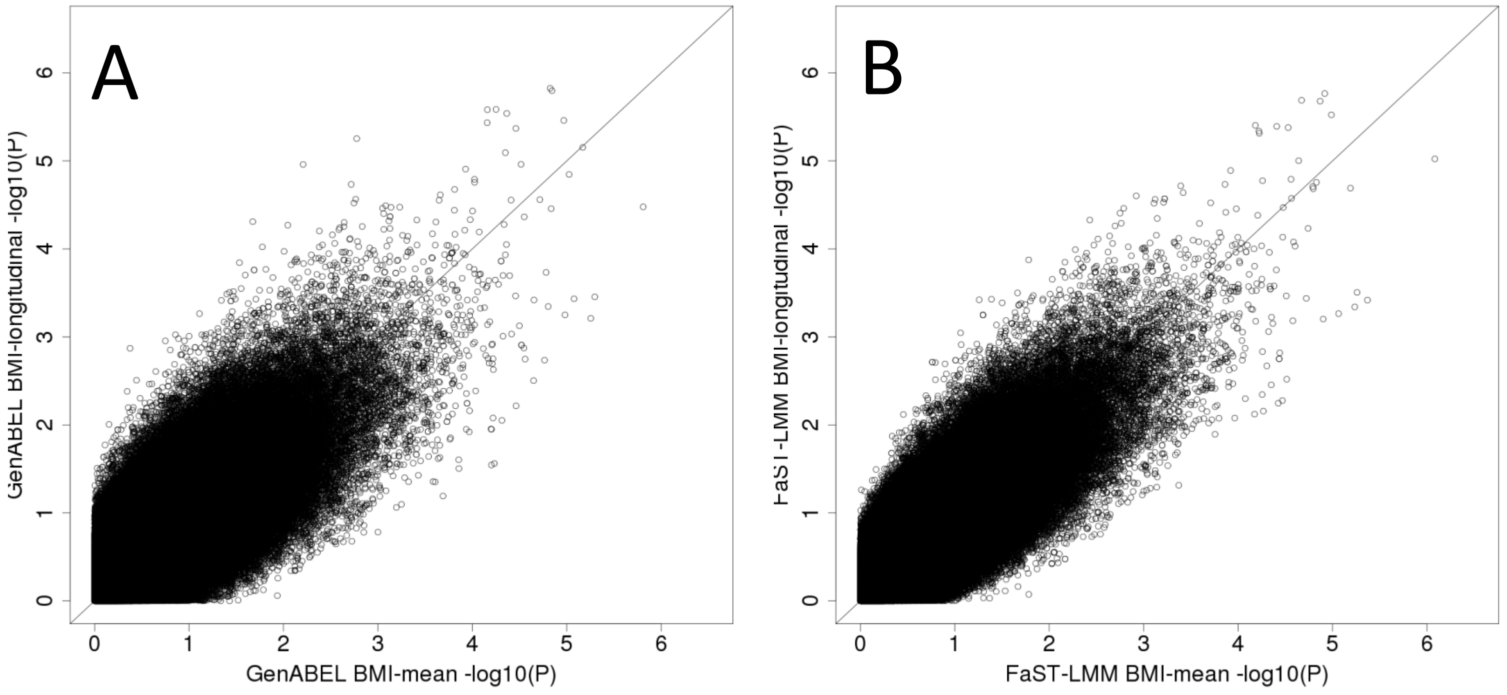

Supplement: S3 Fig — (PDF) [file pone.0119333.s003.pdf]
