# Supplementary material for: First Genome-Wide Association Study in an Australian Aboriginal Population Provides Insights into Genetic Risk Factors for Body Mass Index and Type 2 Diabetes
Source: PLoS One. 2015 Mar 11;10(3):e0119333. doi: 10.1371/journal.pone.0119333 (PMC4356593; doi:10.1371/journal.pone.0119333)

Figure S4. Demonstrates the strongly correlated GWAS results (genotyped data) for FASTA GenABEL versus Fast-LMM for (A) mean BMI, and (B) BMI longitudinal.

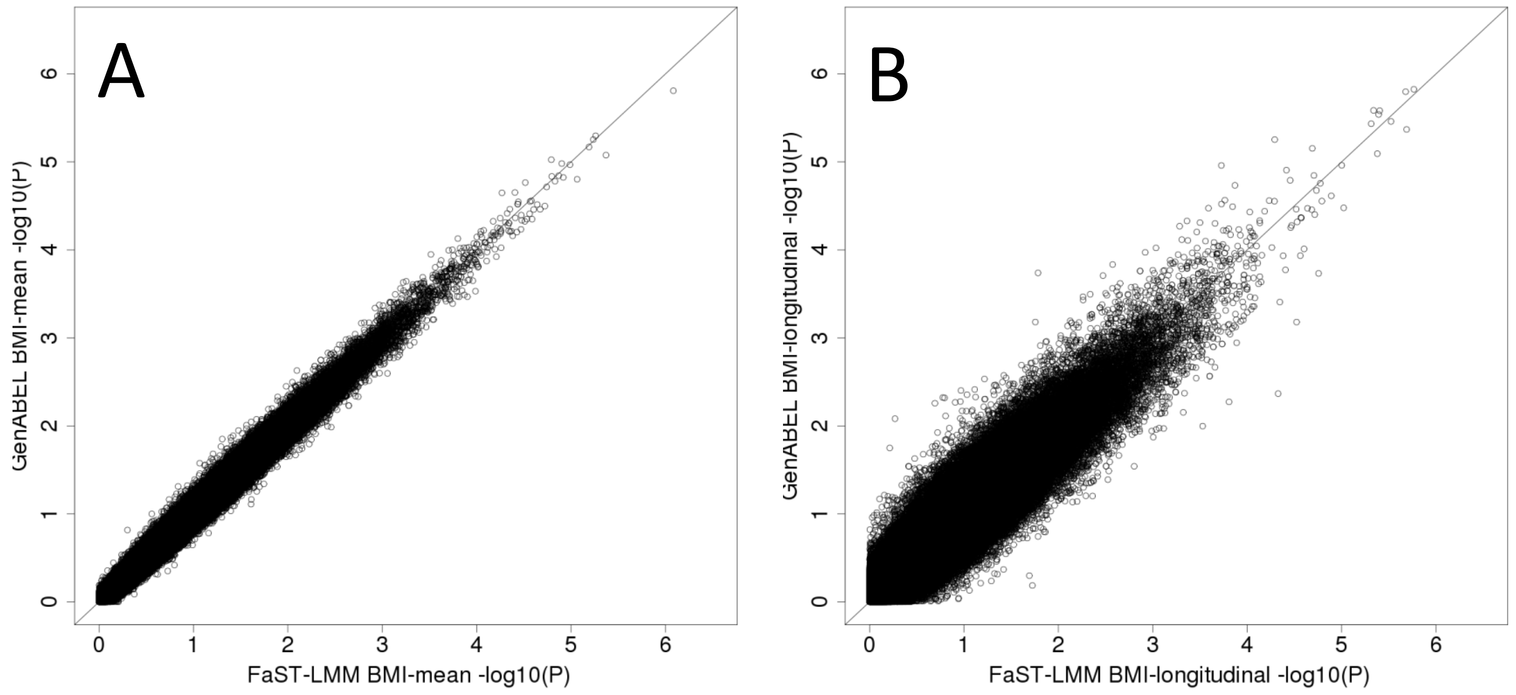

Supplement: S4 Fig — (PDF) [file pone.0119333.s004.pdf]
