# Supplementary material for: First Genome-Wide Association Study in an Australian Aboriginal Population Provides Insights into Genetic Risk Factors for Body Mass Index and Type 2 Diabetes
Source: PLoS One. 2015 Mar 11;10(3):e0119333. doi: 10.1371/journal.pone.0119333 (PMC4356593; doi:10.1371/journal.pone.0119333)

Figure S5. Provides Q-Q plots and associated lambda values for GWAS analyses undertaken for (A) FASTA GenABEL BMI longitudinal, (B) FASTA GenABEL mean BMI, (C) Fast-LMM BMI longitudinal, (D) Fast-LMM mean BMI.

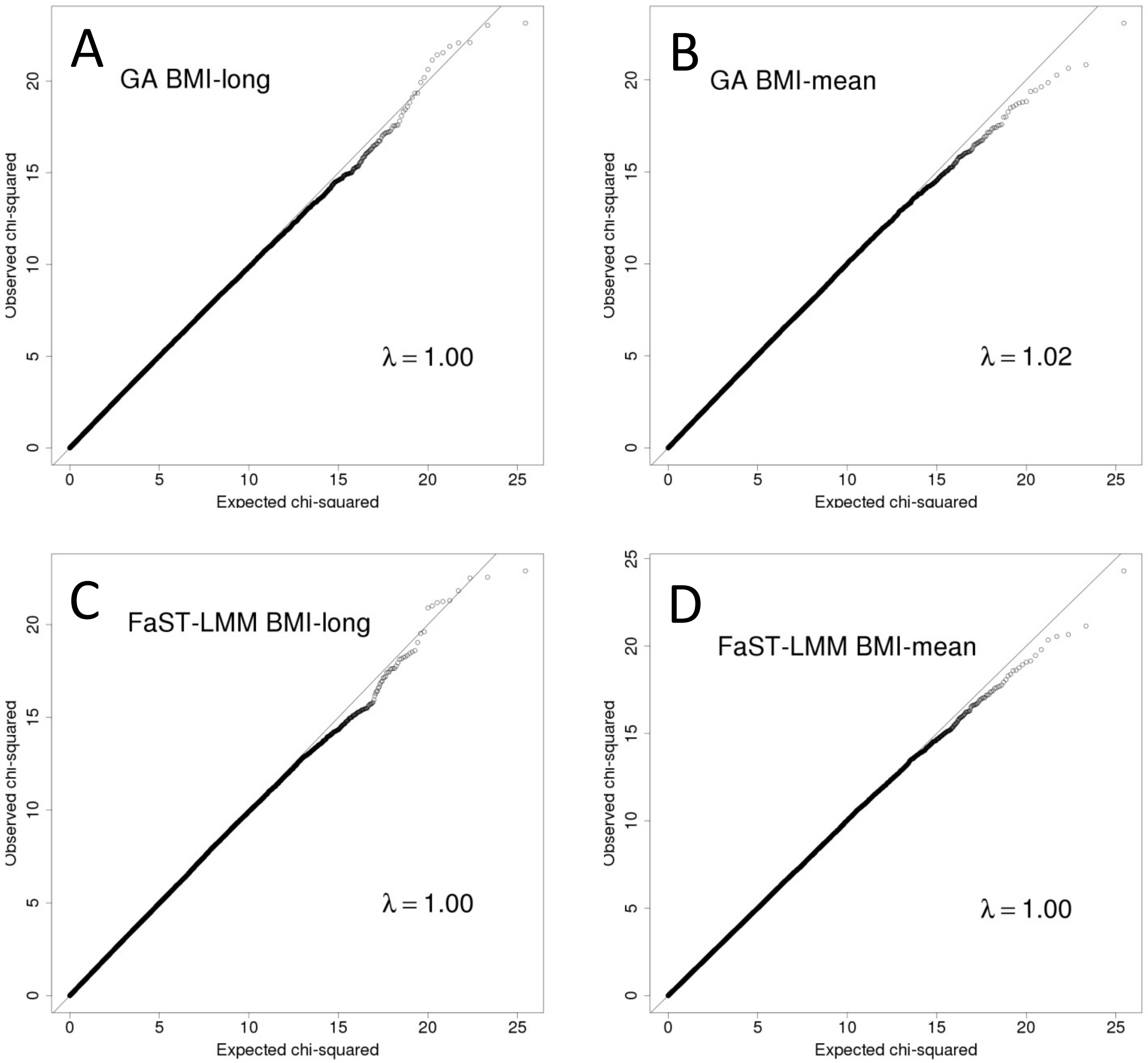

Supplement: S5 Fig — (PDF) [file pone.0119333.s005.pdf]
