# Supplementary material for: First Genome-Wide Association Study in an Australian Aboriginal Population Provides Insights into Genetic Risk Factors for Body Mass Index and Type 2 Diabetes
Source: PLoS One. 2015 Mar 11;10(3):e0119333. doi: 10.1371/journal.pone.0119333 (PMC4356593; doi:10.1371/journal.pone.0119333)

Figure S6. Manhattan plots of genome-wide association results for T2D undertaken using FASTA in GenABEL: (A) results for genotyped data; and (B) results for 1000G imputed data. SNPs in red show the region of the top association in this discovery GWAS.

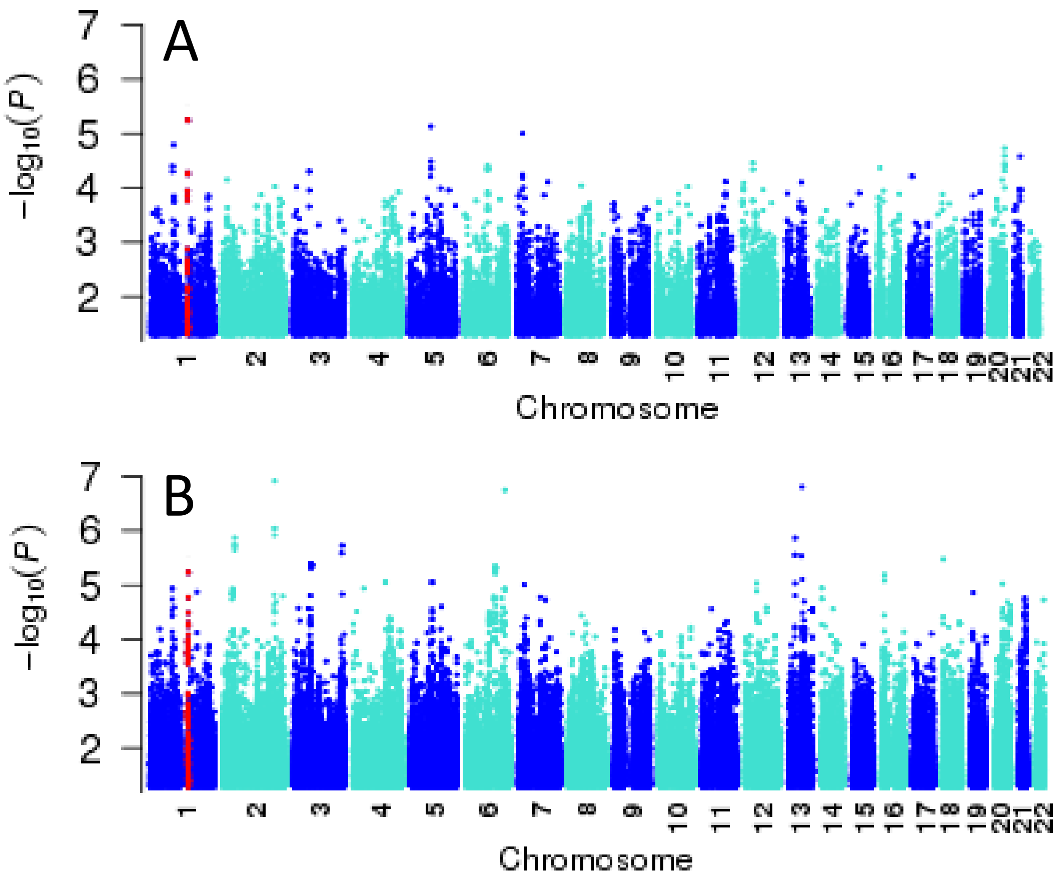

Supplement: S6 Fig — (A) results for genotyped data; and (B) results for 1000G imputed data. SNPs in red show the region of the top association in this discovery GWAS. (PDF) [file pone.0119333.s006.pdf]
