# Supplementary material for: First Genome-Wide Association Study in an Australian Aboriginal Population Provides Insights into Genetic Risk Factors for Body Mass Index and Type 2 Diabetes
Source: PLoS One. 2015 Mar 11;10(3):e0119333. doi: 10.1371/journal.pone.0119333 (PMC4356593; doi:10.1371/journal.pone.0119333)

Figure S7. Regional association plots (Locuszoom) of the imputed SNP signals for BMI (upper graph) and T2D (lower graph) in the regions: (A) *SLC28A3* to *NTRK2* on Chromosome 9; (B) *CNTNAP2* on Chromosome 7; (C) *RBM7* on Chromosome 4; and (D) *PIK3C2G* on Chromosome 12. In each plot the $-\log_{10}$ *P*-values are shown on the upper section, with SNPs colored (see key) based on their r^2 with the labeled top hit SNP (purple), calculated in the 146 unrelated genotyped individuals. Red arrows highlight the position of the BMI hit on the T2D plot. The bottom section of each plot shows the genes marked as horizontal lines.

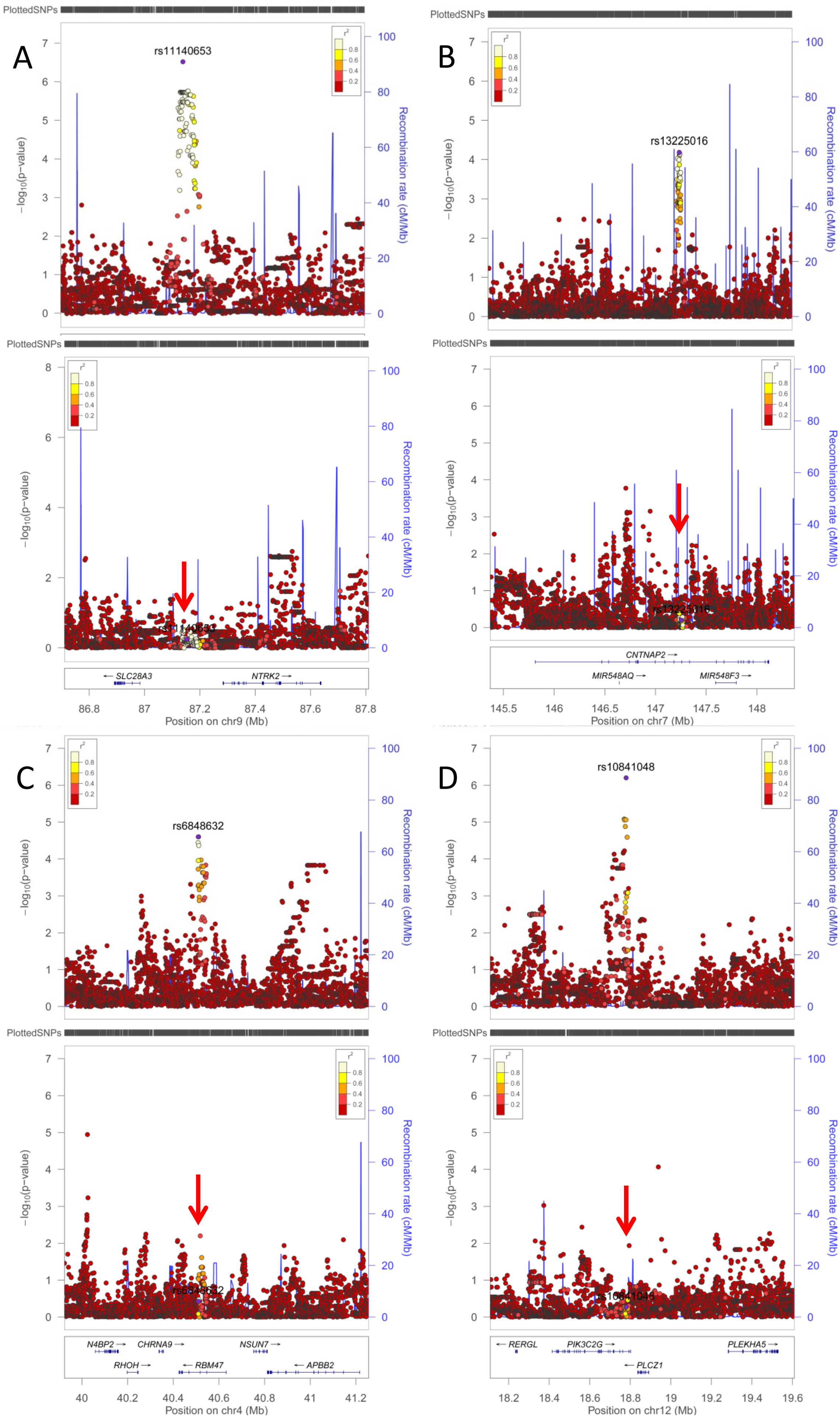

Supplement: S7 Fig — (A) SLC28A3 to NTRK2 on Chromosome 9; (B) CNTNAP2 on Chromosome 7; (C) RBM7 on Chromosome 4; and (D) PIK3C2G on Chromosome 12. In each plot the −log10 P-values are shown on the upper section, with SNPs colored (see key) based on their r2 with the labeled top hit SNP (purple), calculated in the 146 unrelated genotyped individuals. Red arrows highlight the position of the BMI hit on the T2D plot. The bottom section of each plot shows the genes marked as horizontal lines. (PDF) [file pone.0119333.s007.pdf]
