# Supplementary material for: First Genome-Wide Association Study in an Australian Aboriginal Population Provides Insights into Genetic Risk Factors for Body Mass Index and Type 2 Diabetes
Source: PLoS One. 2015 Mar 11;10(3):e0119333. doi: 10.1371/journal.pone.0119333 (PMC4356593; doi:10.1371/journal.pone.0119333)

Figure S8. Regional association plots (Locuszoom[32]) of the signals for (A) BMI genotyped and (B) BMI imputed in the region *SLC28A3* to *NTRK2* on Chromosome 9 before (upper graph) and after (lower graph) conditioning on the top SNPs rs1347857 and rs11140653, respectively. Analysis undertaken using an additive model in FaST-LMM.

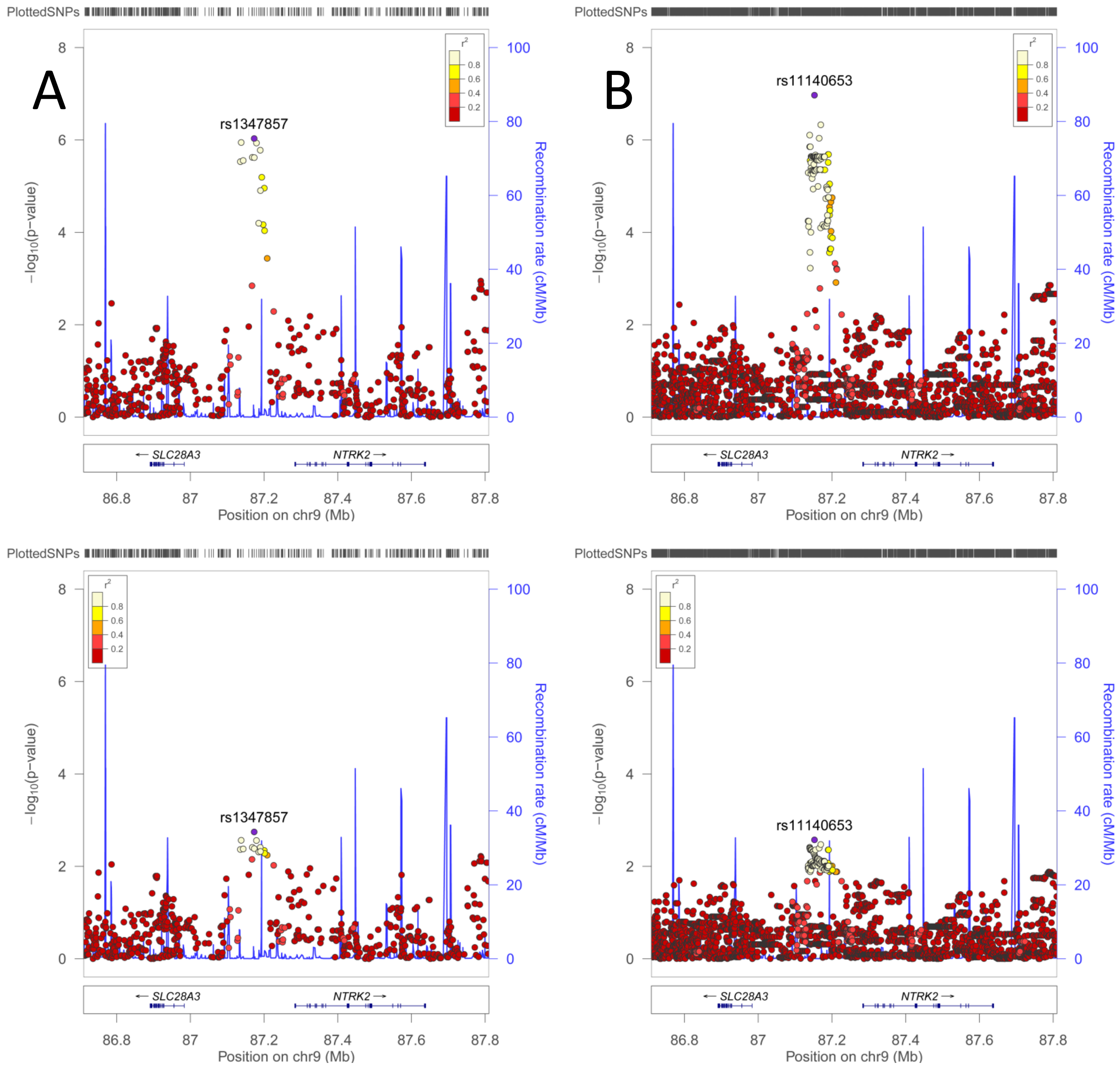

Supplement: S8 Fig — Analysis undertaken using an additive model in FaST-LMM. (PDF) [file pone.0119333.s008.pdf]
