# Supplementary material for: First Genome-Wide Association Study in an Australian Aboriginal Population Provides Insights into Genetic Risk Factors for Body Mass Index and Type 2 Diabetes
Source: PLoS One. 2015 Mar 11;10(3):e0119333. doi: 10.1371/journal.pone.0119333 (PMC4356593; doi:10.1371/journal.pone.0119333)

Figure S9. Plot generated in the UCSC genome-browser for a section of the intergenic region (NCBI Build 37: 87,140,000bp to 87,190,000bp) ~94kb upstream of *NTRK2* on Chromosome 9q21.33. The plot shows positions of the top (P<5x10^{-6}) genotyped (in red vertical lines) and 1000G imputed (in blue vertical lines) SNPs, with the top imputed SNP (rs1140653), the top genotyped SNP (rs1347857) and the SNP of functional interest discussed in the main text (rs1866439) shown again on separate rows. These SNPs are shown relative to: (i) the CpG island-like motif identified using CpG Island Searcher; (ii) the various fragments of LINE-1 (L1) elements (with the L1MA3 element in which the top imputed SNP is located shown in blue); and (iii) peaks of histone methylation (H3K4Me1, H3K4Me3) or acetylation (H3K27Ac) measured in 7 cell lines from the ENCODE project.

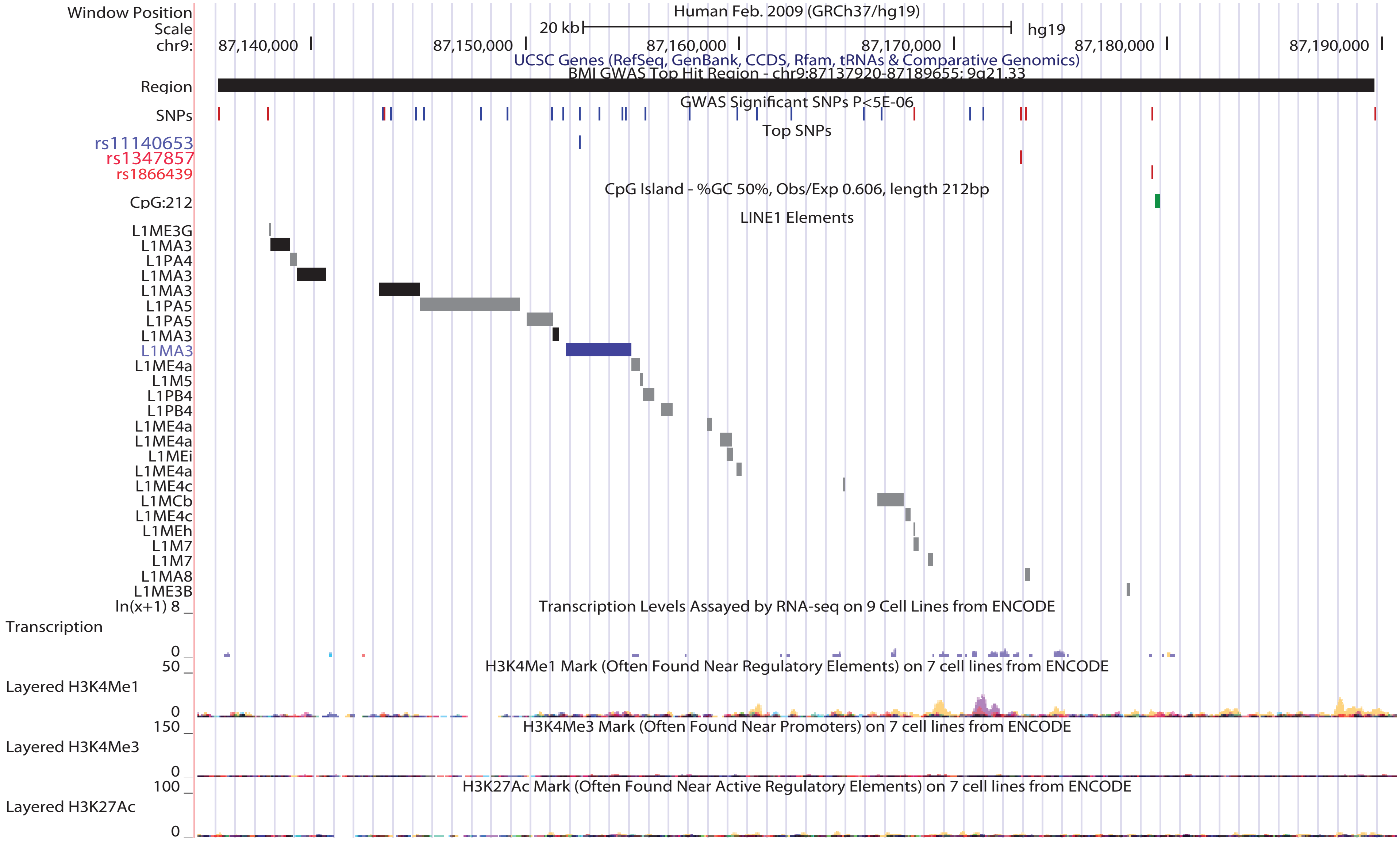

Supplement: S9 Fig — The plot shows positions of the top (P<5×10^-6^) genotyped (in red vertical lines) and 1000G imputed (in blue vertical lines) SNPs, with the top imputed SNP (rs1140653), the top genotyped SNP (rs1347857) and the SNP of functional interest discussed in the main text (rs1866439) shown again on separate rows. These SNPs are shown relative to: (i) the CpG island-like motif identified using CpG Island Searcher; (ii) the various fragments of LINE-1 (L1) elements (with the L1MA3 element in which the top imputed SNP is located shown in blue); and (iii) peaks of histone methylation (H3K4Me1, H3K4Me3) or acetylation (H3K27Ac) measured in 7 cell lines from the ENCODE project. (PDF) [file pone.0119333.s009.pdf]
