# Supplementary material for: First Genome-Wide Association Study in an Australian Aboriginal Population Provides Insights into Genetic Risk Factors for Body Mass Index and Type 2 Diabetes
Source: PLoS One. 2015 Mar 11;10(3):e0119333. doi: 10.1371/journal.pone.0119333 (PMC4356593; doi:10.1371/journal.pone.0119333)
Supplement: S1 Table — The 391 post-QC genotyped individuals used in the GWAS belonged to a small number of interrelated extended pedigrees, as depicted in the radial plot of pairwise identity-by-descent allele-sharing presented in S1 Fig. Characteristics of the 391 genotyped individuals used in the GWAS are provided here. (PDF) [file pone.0119333.s010.pdf]

Supplementary Table 1

Table S1. Characteristics of the family-based Aboriginal study sample. The 391 post-QC genotyped individuals used in the GWAS belonged to a small number of inter-related extended pedigrees, as depicted in the radial plot of pairwise identity-by-descent allele-sharing presented in Figure S1. Characteristics of the 391 genotyped individuals used in the GWAS are provided here.

	Male	Female	Total
Total Sample	179	212	391
N^{o} T2D cases	30	59	89
Age at collection T2D cases			
Mean (SD)	46.93 (12.18)	46.24 (14.51)	46.47 (13.70)
Range	22.22-68.93	16.66-77.47	16.66-77.47
Age at diagnosis T2D cases			
Mean (SD)	41.44 (11.08)	40.30 (14.78)	40.68 (13.62)
Range	20.04-62.89	16.78-76.31	16.78-76.31
Mean BMI T2D cases			
N^{o} with BMI data	30	59	89
Mean (SD)	30.83 (6.66)	33.91 (6.77)	32.87 (6.86)
Range	21.00-42.20	18.33-55.46	18.33-55.46
N^{o} unaffected	149	153	302
Age at collection all unaffected			
Mean (SD)	19.29 (18.61)	17.71 (11.81)	18.49 (15.53)
Range	0.78-80.40	0.13-53.50	0.13-80.40
Age at collection unaffected <20 years of age			
N^{o}	101	95	196
Mean (SD)	9.44 (4.93)	10.05 (5.36)	9.74 (5.14)
Range	0.78-19.99	0.13-19.13	0.13-19.99
Age at collection unaffected ≥20 years of age			
N^{o}	48	58	106
Mean (SD)	40.03 (19.77)	30.25 (8.15)	34.68 (15.53)
Range	21.09-80.40	20.18-53.50	20.18-80.40
Mean BMI all unaffected[a]			
N^{o} with BMI data	135	137	272
Mean (SD)	20.20 (6.11)	23.22 (8.44)	21.72 (7.51)
Range	10.40-44.78	12.43-53.57	10.40-53.57
Mean BMI unaffected <20 years of age			
N^{o} with BMI data	90	86	176
Mean (SD)	17.43 (4.24)	19.35 (5.32)	18.37 (4.88)
Range	10.40-37.95	12.43-32.90	10.40-37.95
Mean BMI unaffected[a] ≥20 years of age			
N^{o} with BMI data	45	51	96
Mean (SD)	25.75 (5.49)	29.74 (8.73)	27.87 (7.62)
Range	17.45-44.78	17.25-53.57	17.25-53.57

[a] Excluding unaffected female BMI outlier shown in Figure 1.
