# Supplementary material for: First Genome-Wide Association Study in an Australian Aboriginal Population Provides Insights into Genetic Risk Factors for Body Mass Index and Type 2 Diabetes
Source: PLoS One. 2015 Mar 11;10(3):e0119333. doi: 10.1371/journal.pone.0119333 (PMC4356593; doi:10.1371/journal.pone.0119333)
Supplement: S2 Table — Bold indicates a SNP hits observed in the imputed but not the genotyped data, including at DICER (P <10-6). (PDF) [file pone.0119333.s011.pdf]

Supplementary Table 2.

Table S2. SNP asociations at $P<0.01$ observed in the WA Aboriginal study population for genes previously reported* to achieve $P<5\times10^{-8}$ (or $P<10^{-5}$ shaded grey) for association with BMI in other populations. Bold indicates a SNP hits observed in the imputed but not the genotyped data, including at at *DICER* ($P<10^{-6}$).

Chromosome			Build 37			BMI	BMI
**Number**	**Gene(s)**	**SNP**	**Bp Position**	**effB**	**se_effB**	**Genotyped**	**1000G Imputed**
1	NTNG1	rs10494072	108,218,139	0.31	0.11	7.27E-03	-
1	NEGR1	rs7535539	72,913,499	-0.32	0.10	1.53E-03	-
1	ATG4C	-	-			-	-
1	AGRN - RNF223	rs2710888	959,842	0.29	0.09	7.23E-04	-
1	SEC16B	-	-			-	-
1	LYPLAL1	rs17580366	219,316,796	-1.07	0.29		2.31E-04
1	LYPLAL1	rs9308387	219,337,294	-0.56	0.18	1.30E-03	
1	TBCE	-	-			-	-
2	ADCY3, DNAJC27	-	-			-	-
2	RBJ, ADCY3, POMC	-	-			-	-
2	TMEM18	-	-			-	-
2	LRP2	rs12614394	170,208,668	0.55	0.20	5.91E-03	-
2	MSTN - C2orf88	-	-			-	-
**2**	**KCNE4**	**rs877302**	**223,667,291**	**1.15**	**0.32**	**-**	**3.96E-04**
2	RPL23P5 - HSPA9P1	rs13424672	222,827,492	0.57	0.18	1.72E-03	-
2	IRS1	rs1560251	227,653,948	-0.29	0.09	1.98E-03	
2	IRS1	rs1801123	227,661,043	-0.32	0.10	-	8.37E-04
2	LRRFIP1	-	-			-	-
3	UQCRHP4 - HMGB1P36	rs8179901	71,024,676	0.29	0.09	1.93E-03	-
3	CCK	rs34259319	42,445,871	-1.23	0.35	-	4.08E-04
3	CCK	rs2278215	42,555,524	0.30	0.09	1.41E-03	
3	VGLL4	rs17778035	11,677,695	0.29	0.11	9.85E-03	-
3	PP13439, TMEM212	-	-			-	-
3	SFRS10, ETV5, DGKG	-	-			-	-
3	LPP	rs74698002	187,942,700	-0.31	0.11	7.72E-05	-
4	GNPDA2	-	-			-	-
4	LINC00290	rs4362863	181,985,824	-0.64	0.19	-	8.57E-04
4	LINC00290	rs13120437	182,065,793	-0.33	0.10	1.39E-03	-
5	CDH9	rs79838142	26,784,102	1.66	0.46	-	2.70E-04
5	CDH9	rs16896091	26,785,851	0.57	0.19	2.60E-03	-
5	ROPN1L	rs6883094	10,512,454	-0.42	0.15	4.16E-03	-
5	MARCH6	rs6883094	10,512,454	-0.42	0.15	4.16E-03	-
5	SSBP2	rs1542596	81,247,381	-0.24	0.09	8.09E-03	-
5	PCSK1	rs199880258	95,924,814	0.35	0.09	2.55E-04	-
5	PCSK1	rs9314174	95,925,508	0.36	0.10	-	1.89E-04
5	MFAP3, GALNT10	rs1461229	153,257,636	-0.36	0.10	3.50E-04	
5	MFAP3, GALNT10	rs1461229	153,257,636	-0.36	0.10		4.01E-04
5	OR2Y1 - MGAT1	rs17703464	180,265,135	0.36	0.13	4.88E-03	-
6	TFAP2B	-	-			-	-
6	CDKAL1	-	-			-	-
6	LAMA2	-	-			-	-
6	SGK1, LOC442261	-	-			-	-
**7**	**ZNF804B**	**rs143130157**	**88,464,147**	**0.98**	**0.28**	**-**	**4.86E-04**
7	RPS15AP22 - PLXNA4	-	-			-	
7	CNTNAP2	rs13225016	147,256,110	-0.35	0.09	-	6.57E-05
7	CNTNAP2	rs6960319	147,258,631	-0.36	0.09	6.88E-06	
7	CNTNAP2	rs6960319	147,258,631	**-0.35**	**0.09**		7.26E-05
8	MSRA	-	-			-	-
8	MMP16	-	-			-	-
**8**	**TRHR**	**rs76336427**	**110,377,338**	**-1.25**	**0.31**	**-**	**4.50E-05**

Supplementary Table 2.

Chromosome			Build 37			BMI	BMI
Number	Gene(s)	SNP	Bp Position	effB	se_effB	Genotyped	1000G Imputed
9	RNU6-14 - BNC2	-	-			-	-
9	LOC347097, PIP5K1B	-	-			-	-
9	KLF9	-	-			-	-
9	ZNF169	-	-			-	
9	RAD23B	rs7023656	110,075,437	-0.33	0.10	5.24E-04	
9	RAD23B	rs7023656	110,075,437	-0.32	0.10		8.54E-04
10	KLF6 - LINC00705	rs3829203	3,858,226	0.30	0.09	7.44E-04	
10	KLF6 - LINC00705	rs3829203	3,858,226	0.30	0.09		9.48E-04
11	MTCH2	-	-			-	-
11	PAX6	-	-			-	-
11	BDNF	-	-			-	-
11	STK33	-	-			-	-
11	GLYAT	-	-			-	-
11	ARL6IP1P3 - MIR4300	-	-			-	-
12	CHCHD3P2 - RPL31P48	-	-			-	-
12	ANKS1B	-	-			-	-
13	STARP1 - HNRNPA3P5	-	-			-	-
13	SPRY2	-	-			-	-
13	FAM155A	-	-			-	-
14	AKAP6	-	-			-	-
14	LOC645687, PELI2	-	-			-	-
**14**	**RPL15P2, DICER1**	**rs12897644**	**95,838,401**	**1.54**	**0.31**	**-**	**8.61E-07**
14	LOC100128373, DIO3OS	-	-			-	-
15	MAP2K5	rs74606175	68,219,455	0.91	0.26	-	5.14E-04
16	SH2B1	-	-			-	-
16	GP2	-	-			-	-
16	GPRC5B, IQCK	-	-			-	
16	FTO	rs16953231	54,262,571	0.69	0.20	-	7.88E-04
17	SLC39A11	rs2567503	70,713,365	-1.11	0.31	-	3.18E-04
17	SEPT9	-	-			-	-
18	MC4R	rs12959775	58,533,764	-0.48	0.13	4.49E-04	
19	AP3D1 - DOT1L	rs117734617	2,193,653	-1.36	0.41	-	8.45E-04
19	KCTD15, CHST8	-	-			-	-
19	KCTD15	-	-			-	-
19	GIPR	-	-			-	-
19	GIPR, QPCTL	-	-			-	-
19	SIGLEC23P - SIGLEC24P	-	-			-	-
20	CSRP2BP	rs35183839	18,137,412	0.57	0.17	-	9.66E-04
20	BMP2	-	-			-	-
21	GRIK1	-	-			-	-

* As reported in the NHGRI GWAS Catalog: Hindorff LA, MacArthur J (European Bioinformatics Institute), Morales J (European Bioinformatics Institute), Junkins HA, Hall PN, Klemm AK, and Manolio TA. A Catalog of Published Genome-Wide Association Studies. Available at: www.genome.gov/gwastudies. Accessed 26 April 2014. Dash indicates P>0.01 in our study.
