# Supplementary material for: First Genome-Wide Association Study in an Australian Aboriginal Population Provides Insights into Genetic Risk Factors for Body Mass Index and Type 2 Diabetes
Source: PLoS One. 2015 Mar 11;10(3):e0119333. doi: 10.1371/journal.pone.0119333 (PMC4356593; doi:10.1371/journal.pone.0119333)
Supplement: S3 Table — Results for the top 50 hits for “BMI longitudinal” GWAS analysis in GenABEL using each individual BMI reading as a separate observation, modelling the correlation between readings via the estimated kinship and using the genomic control deflation factor to avoid inflation of the overall distribution of test statistics. Results are for allele-wise tests under an additive model of inheritance. Bold indicates SNP associations of functional interest presented in main Table 1. (PDF) [file pone.0119333.s012.pdf]

Supplementary Table 3

Table S3. Top GWAS autosomal SNP hits for BMI, organised by chromosome. Results for the top 50 hits for "BMI longitudinal" GWAS analysis in GenABEL using each individual BMI reading as a separate observation, modelling the correlation between readings via the estimated kinship and using the genomic control deflation factor to avoid inflation of the overall distribution of test statistics. Results are for allele-wise tests under an additive model of inheritance adjusting for sex and age[5]. Bold indicates SNP associations of functional interest presented in main Table 1.

Chromosome	SNP	NCBI37	A1	A2	effB	se_effB	$P_{deflated}$	SNP Location	HGNC*
2	rs12996522	2523844	G	C	-0.71	0.18	4.69E-05	INTERGENIC	MYT1L/-
2	rs4972533	174070885	A	C	0.70	0.17	5.74E-05	INTRONIC	MLTK
2	rs3769157	174079402	A	G	0.70	0.17	6.04E-05	INTRONIC	MLTK
2	rs6707969	174105869	C	A	0.63	0.15	5.01E-05	INTRONIC	MLTK
3	rs9829757	154800995	A	G	0.38	0.09	4.32E-05	INTRONIC	MME
4	rs1378233	12328063	A	G	0.36	0.09	6.42E-05	INTERGENIC	-
**4**	**rs6848632**	**40509521**	**A**	**C**	**0.52**	**0.12**	**1.43E-05**	**INTRONIC**	**RBM47**
4	rs61698614	40510828	G	A	0.49	0.12	3.49E-05	INTRONIC	RBM47
4	rs2250361	179391633	A	G	-0.63	0.16	6.81E-05	INTERGENIC	-
4	rs12641600	186839888	G	A	-0.37	0.09	2.75E-05	INTRONIC	SORBS2
5	rs13171074	1782838	G	A	0.36	0.09	5.56E-05	INTERGENIC	SDHAP3/MRPL36
7	rs4728956	90788209	G	A	-0.38	0.09	3.23E-05	INTRONIC	CDK14
7	rs6465304	90818255	G	A	-0.36	0.09	6.00E-05	INTRONIC	CDK14
**7**	**rs6960319**	**147258631**	**G**	**A**	**-0.36**	**0.09**	**4.65E-05**	**INTRONIC**	**CNTNAP2**
7	rs13236486	147262724	A	G	-0.35	0.09	6.12E-05	INTRONIC	CNTNAP2
8	rs2554653	3793600	A	G	0.41	0.10	3.71E-05	INTRONIC	CSMD1
8	rs7017291	54759323	C	A	-0.52	0.13	4.90E-05	UPSTREAM	ATP6V1H
9	rs11144217	77696677	G	A	-0.39	0.09	3.45E-05	INTRONIC	NMRK1
9	rs7871278	87135644	A	G	0.42	0.09	3.68E-06	INTERGENIC	SLC28A3/NTRK2
9	rs11140640	87137920	G	A	0.42	0.09	4.27E-06	INTERGENIC	SLC28A3/NTRK2
9	rs1926746	87143351	C	A	0.44	0.09	2.60E-06	INTERGENIC	SLC28A3/NTRK2
9	rs2068225	87168094	A	G	0.43	0.09	2.62E-06	INTERGENIC	SLC28A3/NTRK2
**9**	**rs1347857**	**87173097**	**A**	**C**	**0.44**	**0.09**	**1.50E-06**	**INTERGENIC**	**SLC28A3/NTRK2**
9	rs1347858	87173333	C	A	0.44	0.09	2.89E-06	INTERGENIC	SLC28A3/NTRK2
9	rs1866439	87179264	G	A	0.44	0.09	1.59E-06	INTERGENIC	SLC28A3/NTRK2
9	rs10868204	87189655	C	A	0.43	0.09	3.47E-06	INTERGENIC	SLC28A3/NTRK2
9	rs11140676	87190178	G	A	0.40	0.09	2.11E-05	INTERGENIC	SLC28A3/NTRK2
9	rs2118819	87193728	G	A	0.42	0.10	1.09E-05	INTERGENIC	SLC28A3/NTRK2
9	rs2218090	87200556	A	G	0.40	0.10	2.79E-05	INTERGENIC	SLC28A3/NTRK2
9	rs1961628	93862393	G	A	0.44	0.11	4.94E-05	INTERGENIC	SYK/AUH
10	rs11239187	45133277	A	G	-0.56	0.12	5.58E-06	INTERGENIC	CXCL12/TMEM72
10	rs11239189	45134563	A	G	-0.50	0.13	6.26E-05	INTERGENIC	CXCL12/TMEM72
12	rs11044192	18724543	A	G	0.42	0.11	6.92E-05	INTRONIC	PIK3C2G
12	rs11837598	18765013	G	A	0.44	0.11	5.58E-05	INTRONIC	PIK3C2G
**12**	**rs12816270**	**18770577**	**A**	**G**	**0.72**	**0.16**	**8.06E-06**	**INTRONIC**	**PIK3C2G**
12	rs4764409	18784252	G	A	0.67	0.16	2.43E-05	INTRONIC	PIK3C2G
12	rs59392591	114326282	A	G	-0.51	0.13	5.87E-05	INTRONIC	RBM19
13	rs2151436	27894047	G	A	0.40	0.09	1.75E-05	UPSTREAM	AL159977.1
13	rs12865097	45477410	G	A	0.78	0.17	7.02E-06	INTERGENIC	LINC003300/NUFIP1
15	rs57758826	97312049	A	G	0.50	0.12	3.97E-05	DOWNSTREAM	SPATA8
15	rs66883979	97316477	G	A	0.51	0.12	3.37E-05	DOWNSTREAM	SPATA8
17	rs16959542	16058111	A	G	0.50	0.12	4.30E-05	INTRONIC	NCOR1
17	rs115845128	16146300	G	A	0.49	0.12	4.81E-05	INTRONIC	PIGL
17	rs3112512	16185122	G	A	0.50	0.12	4.30E-05	INTRONIC	PIGL
18	rs1791161	29204210	A	G	-0.40	0.09	1.24E-05	3_PRIME_UTR	B4GALT6
18	rs1026123	29207155	A	G	-0.37	0.09	3.64E-05	INTRONIC	B4GALT6
18	rs1667280	29219172	A	G	-0.36	0.09	6.73E-05	INTRONIC	B4GALT6
18	rs1667284	29226208	G	A	-0.39	0.09	1.62E-05	INTRONIC	B4GALT6
20	rs7260870	11879084	G	A	-0.39	0.10	5.28E-05	INTRONIC	BTBD3
20	rs74723987	19983850	A	G	0.52	0.13	3.33E-05	DOWNSTREAM	RIN2

* Genes separated by comma indicate SNP is within both genes; genes separated by forward slash indicate genes within 250 kb upstream/downstream of the SNP; dash indicates large intergenic region with no protein coding genes within 250 kb on either side of the SNP. NCBI37 = bp location on chromosome for NCBI Build 37. A1 = major allele; A2 = minor allele.
