# Supplementary material for: First Genome-Wide Association Study in an Australian Aboriginal Population Provides Insights into Genetic Risk Factors for Body Mass Index and Type 2 Diabetes
Source: PLoS One. 2015 Mar 11;10(3):e0119333. doi: 10.1371/journal.pone.0119333 (PMC4356593; doi:10.1371/journal.pone.0119333)
Supplement: S4 Table — Bold indicates SNP hits observed in the imputed but not the genotyped data, including at IGF2BP2 (P <10-5). (PDF) [file pone.0119333.s013.pdf]

Table S4. SNP associations observed at nominal P<0.01 in the WA Aboriginal study population for genes previously reported to achieve *P* <5x10^{-8} for association with T2D in other populations. Bold indicates SNP hits observed in the imputed but not the genotyped data, including at *IGF2BP2* (*P* <10-5) .

Chromosome Number	Gene By Functional Group	SNP	Build 37 Bp Position	effB	se_effB	T2D Genotyped	T2D 1000G Imputed
Insulin Secretion Beta cell Dysfunction							
2	THADA	rs2288709	44,004,010	0.09	0.03	7.52E-03	-
3	ADCY5	rs35519015	123,027,926	-0.14	0.05	9.06E-03	-
**3**	**IGF2BP2**	**rs138306797**	**185,545,719**	**0.78**	**0.16**	**-**	**2.55E-06**
4	WFS1	-	-	-	-	-	-
7	DGKB	-	-	-	-	-	-
**7**	**JAZF1**	**rs4722801**	**28,523,547**	**0.36**	**0.09**	**-**	**1.11E-04**
7	GCK	-	-	-	-	-	-
8	ANK1	rs10097592	41,261,544	-0.11	0.03	8.74E-04	
8	ANK1	rs10097592	41,261,544	-0.11	0.03		8.74E-04
8	SLC30A8	-	-	-	-	-	-
10	CDC123/CAMK1D	rs7895410	12,310,737	0.08	0.03	7.63E-03	-
10	TCF7L2	-	-	-	-	-	-
11	KCNQ1	-	-	-	-	-	-
11	KCNJ11	-	-	-	-	-	-
11	CENTD2	-	-	-	-	-	-
**11**	**MTNR1B(SLC36A4)**	**rs1446911**	**92,932,048**	**0.13**	**0.04**	**-**	**2.26E-04**
12	TSPAN8	rs1580713	71,500,166	-0.10	0.03	6.16E-04	
	TSPAN8	rs1705218	71,511,343	-0.10	0.03		5.91E-04
12	LGR5	rs1148982	71,952,587	-0.10	0.03	2.28E-03	-
15	C2CD4A	-	-	-	-		-
18*	LAMA1	rs648161	7,042,402	-0.10	0.03	4.84E-03	-
19	GIPR	-	-	-	-	-	-
Insulin Secretion Beta cell Development				-	-		
1	PROX1	rs6686424	214,196,634	0.17	0.05	7.48E-04	
1	PROX1	rs10494972	214,207,351	0.19	0.05		3.48E-04
6	CDKAL1	-	-	-	-	-	-
9	GLIS3	rs681410	4,036,730	0.14	0.04	2.56E-04	
9	GLIS3	rs681410	4,036,730	0.14	0.04		2.56E-04
9	CDKN2A/B	-	-	-	-	-	-
10	IDE	-	-	-	-	-	-
10	HHEX	-	-	-	-	-	-
12	HNF1A	-	-	-	-	-	-
17	HNF1B	-	-	-	-	-	-
20	HNF4A	-	-	-	-	-	-
Insulin Resistance Obesity				-	-		
2	GRB14	-	-	-	-	-	-
7	KLF14	-	-	-	-	-	-
**10***	**GRK5**	**rs1810158**	**120,955,500**	**0.28**	**0.09**	-	**9.54E-04**
**13**	**SPRY2**	**rs12873997**	**80,853,383**	**0.15**	**0.04**	-	**1.99E-05**
16	FTO	-	-	-	-	-	-
**18**	**MC4R**	**rs184454385**	**58,269,728**	**0.39**	**0.12**	-	**8.64E-04**
Insulin Resistance Insulin Action				-	-		
2	GCKR	-	-	-	-	-	-
2	IRS1	-	-	-	-	-	-
**3**	**PPARG**	**rs73027292**	**12,492,062**	**0.48**	**0.13**	-	**3.18E-04**
**3**	**(PRICKLE2)ADAMTS9**	**rs10490791**	**64,316,282**	**0.54**	**0.13**	-	**3.80E-05**
5	ANKRD55	-	-	-	-	-	-

Chromosome	Gene		Build 37			T2D	T2D
Number	By Functional Group	SNP	Bp Position	effB	se_effB	Genotyped	1000G Imputed
**Unknown**							
1	NOTCH2	-	-	-	-	-	-
2	BCL11A	-	-	-	-	-	-
**2***	**RND3/RBM43**	**rs983231**	**151,205,806**	**0.19**	**0.05**	**-**	**1.34E-04**
2	RBMS1	-	-	-	-	-	-
3	UBE2E2	rs60079501	23,218,091	0.13	0.04	9.88E-04	
3	UBE2E2	rs60079501	23,218,091	0.13	0.04		9.88E-04
**3**	**PSMD6**	**rs114015970**	**64,061,521**	**0.60**	**0.14**	**-**	**1.54E-05**
3	ST6GAL1	rs16861319	186,665,015	0.51	0.15	-	5.44E-04
3	ST6GAL1	rs4626092	186,698,993	0.10	0.03	3.98E-03	
4*	CTBP1-AS1/MGC21675	-	-	-	-	-	-
4	MAEA	-	-	-	-	-	-
5	ZBED3	-	-	-	-	-	-
6	ZFAND3	-	-	-	-	-	-
6	KCNK16	-	-	-	-	-	-
7	GCC1	-	-	-	-	-	-
7*	PAX4	-	-	-	-	-	-
8	TP53INP1	-	-	-	-	-	-
9	PTPRD	rs139302873	8,256,027	0.36	0.10	-	4.71E-04
9	PTPRD	rs1072692	9,159,897	0.12	0.04	3.78E-03	-
9	TLE4	-	-	-	-	-	-
**9**	**TLE1**	**rs11139282**	**84,096,951**	**0.50**	**0.14**	**-**	**5.01E-04**
10	VPS26A	-	-	-	-	-	-
10	ZMIZ1	rs719338	80,851,257	-0.10	0.03	1.00E-03	-
11	DUSP8	-	-	-	-	-	-
12	CCND2	-	-	-	-	-	-
12	KLHDC5	-	-	-	-	-	-
12	HMGA2	rs4762124	66,302,865	0.10	0.03	2.58E-03	
**15***	**RASGRP1**	**rs74009831**	**38,845,468**	**0.31**	**0.09**	**-**	**6.24E-04**
15	HMG20A	-	-	-	-	-	-
**15**	**ZFAND6**	**rs185099918**	**80,408,046**	**0.41**	**0.12**	**-**	**5.52E-04**
15	AP3S2	-	-			-	-
**15**	**PRC1**	**rs4331318**	**91,582,257**	**0.14**	**0.04**	**-**	**9.42E-04**
16	BCAR1	-	-	-	-	-	-
17	SRR	-	-	-	-	-	-
19	CILP2	-	-	-	-	-	-
19	PEPD	-	-	-	-	-	-

Genes included have been reported from previous GWAS at $P<5x10^{-8}$, as reported in the NHGRI GWAS Catalog: Hindorff LA, MacArthur J (European Bioinformatics Institute), Morales J (European Bioinformatics Institute), Junkins HA, Hall PN, Klemm AK, and Manolio TA. A Catalog of Published Genome-Wide Association Studies. Available at: www.genome.gov/gwastudies. Accessed 15 May 2013. Genes are listed according to functional categories as reported by Kwak and Park, 2013. * indicates genes added to the catalog since that review was published. Dash indicates P>0.01 in our study population.
