# Supplementary material for: First Genome-Wide Association Study in an Australian Aboriginal Population Provides Insights into Genetic Risk Factors for Body Mass Index and Type 2 Diabetes
Source: PLoS One. 2015 Mar 11;10(3):e0119333. doi: 10.1371/journal.pone.0119333 (PMC4356593; doi:10.1371/journal.pone.0119333)
Supplement: S5 Table — Results are for allele-wise tests under an additive model of inheritance. Bold indicates SNP associations of functional interest presented in main Table 2. (PDF) [file pone.0119333.s014.pdf]

Table S5. Top 50 GWAS SNP hits for T2D, organised by chromosome. Results are for allele-wise tests under an additive model of inheritance. Bold indicates SNP associations of functional interest presented in main Table 2.

Chr	SNP	NCBI37	A1	A2	effB	se_effB	P1df	SNP Location	HGNC*
1	rs17112247	85064272	A	G	0.16	0.04	4.93E-05	INTERGENIC	CTBD/C1orf180,SSX2IP
1	rs2138169	85073039	G	A	0.20	0.05	3.91E-05	INTERGENIC	CTBD/C1orf180,SSX2IP
1	rs12732279	89825320	A	G	0.15	0.04	1.60E-05	UPSTREAM	GBP6
1	rs17160295	146977954	G	A	0.15	0.04	5.55E-05	INTERGENIC	CHD1L/BCL9
1	rs17160305	146981725	G	A	0.15	0.04	5.22E-05	INTERGENIC	CHD1L/BCL9
**1**	**rs11240074**	**146996480**	**A**	**C**	**0.21**	**0.05**	**5.59E-06**	INTERGENIC	**CHD1L/BCL9**
1	rs11240075	146996800	G	A	0.21	0.05	5.61E-06	INTERGENIC	CHD1L/BCL9
2	rs66906084	18597215	C	A	-0.13	0.03	7.04E-05	INTERGENIC	KCNS3/RDH14
2	rs768180	205066703	A	G	-0.13	0.03	9.43E-05	INTERGENIC	ICOS/PARD3B
3	rs4685088	14265936	A	G	0.13	0.03	9.59E-05	INTERGENIC	LSM3/SLC6A6
3	rs1870919	64072378	A	C	0.29	0.07	4.95E-05	INTERGENIC	PSMD6/PRICKLE2
3	rs254856	64072820	G	A	0.29	0.07	4.95E-05	INTERGENIC	PSMD6/PRICKLE2
5	rs2973342	80841753	A	G	0.20	0.05	6.07E-05	INTRONIC	SSBP2
5	rs242061	82003267	A	G	0.22	0.05	4.23E-05	INTERGENIC	ATP6AP1L/TMEM167A,XRCC4
5	rs355289	82007207	C	A	0.22	0.05	4.43E-05	INTERGENIC	ATP6AP1L/TMEM167A,XRCC4
5	rs4703911	82012854	G	A	0.22	0.05	7.37E-06	INTERGENIC	ATP6AP1L/TMEM167A,XRCC4
5	rs4703915	82024026	G	A	0.22	0.05	3.22E-05	INTERGENIC	ATP6AP1L/TMEM167A,XRCC4
5	rs3923326	118516878	G	A	-0.12	0.03	1.01E-04	INTRONIC	DMXL1
6	rs9444673	89882208	G	A	-0.13	0.03	4.65E-05	INTERGENIC	PM20DC/GABRR1
6	rs9451173	89892819	A	C	-0.13	0.03	3.88E-05	INTRONIC	GABRR1
**6**	**rs6930407**	**89905239**	**A**	**G**	**0.13**	**0.03**	**4.55E-05**	INTRONIC	**GABRR1**
7	rs7794890	22412741	A	G	0.15	0.03	9.80E-06	INTERGENIC	RAPGEF5/STEAP1B
7	rs10228562	22416306	G	A	0.15	0.04	6.03E-05	INTERGENIC	RAPGEF5/STEAP1B
7	rs2528851	22424545	G	A	0.15	0.04	5.73E-05	INTERGENIC	RAPGEF5/STEAP1B
7	rs4722136	22431079	G	A	0.15	0.04	6.63E-05	INTERGENIC	RAPGEF5/STEAP1B
7	rs12540380	25674531	A	G	0.15	0.04	9.74E-05	INTRONIC	AC003090.1.1
7	rs2057876	121282782	A	G	0.13	0.03	7.69E-05	INTERGENIC	FAM3C/PTPRZ1
8	rs77382280	60177047	C	A	0.13	0.03	9.16E-05	INTERGENIC	TOX/RNA5SP267
10	rs4615961	123156384	C	A	0.12	0.03	9.46E-05	INTERGENIC	WDR11/FGFR2
11	rs1917448	103498150	A	G	0.13	0.03	7.60E-05	INTERGENIC	DYNC2H1/PDGFD
**12**	**rs11063387**	**4998536**	**G**	**C**	**0.14**	**0.03**	**8.24E-05**	INTERGENIC	**KCNA6/KCNA1**
12	rs57770742	5001319	A	G	0.13	0.03	9.75E-05	INTERGENIC	KCNA6/KCNA1
12	rs1849781	43573966	G	A	0.13	0.03	3.43E-05	INTERGENIC	PRICKLE1/ADAMTS20
12	rs6421254	43580562	G	A	0.13	0.03	4.57E-05	INTERGENIC	PRICKLE1/ADAMTS20
12	rs12320335	82280797	G	A	0.12	0.03	1.07E-04	INTERGENIC	PPFIA2/CCDC59
13	rs9575720	85616541	G	A	0.17	0.04	7.82E-05	INTERGENIC	-
16	rs2868980	11754454	A	G	0.13	0.03	4.27E-05	INTERGENIC	UTAF/SNN,TXNDC11
17	rs112038695	15587089	A	G	-0.22	0.06	6.10E-05	5'_UTR	TRIM16
20	rs60664722	51661874	A	G	0.16	0.04	7.11E-05	INTRONIC	TSHZ2
20	rs6062131	60766599	A	C	0.14	0.03	4.81E-05	INTRONIC	GTPBP5
20	rs6089688	60775339	C	A	0.14	0.03	3.88E-05	INTRONIC	GTPBP5
20	rs2184156	60777171	G	A	0.14	0.03	3.53E-05	INTRONIC	GTPBP5
20	rs11696152	60777929	G	A	0.13	0.03	9.86E-05	INTRONIC	GTPBP5
20	rs1047154	60778208	C	A	0.14	0.03	2.54E-05	INTRONIC	GTPBP5
20	rs58191584	60782293	G	A	0.14	0.03	1.84E-05	DOWNSTREAM	GTPBP5
**21**	**rs8128418**	**39188732**	**G**	**A**	**0.25**	**0.06**	**1.07E-04**	INTRONIC	**KCNJ6**
21	rs11088461	39276350	G	A	0.22	0.06	1.07E-04	INTERGENIC	ETS2/PSMG1,BRWD1
21	rs9982548	40351828	G	A	0.27	0.06	2.62E-05	INTERGENIC	ETS2/PSMG1,BRWD1
21	rs732340	40363690	G	A	0.22	0.06	1.05E-04	INTERGENIC	ETS2/PSMG1,BRWD1
21	rs732339	40363878	C	A	0.22	0.06	1.05E-04	INTERGENIC	ETS2/PSMG1,BRWD1

* Genes separated by comma indicate SNP is within both genes; genes separated by forward slash indicate the nearest protein coding genes upstream/downstream of the SNP; dash indicates large intergenic region with no protein coding genes within 250 kb on either side of the SNP. A1 = major allele; A2 = minor allele.
