# Supplementary material for: First Genome-Wide Association Study in an Australian Aboriginal Population Provides Insights into Genetic Risk Factors for Body Mass Index and Type 2 Diabetes
Source: PLoS One. 2015 Mar 11;10(3):e0119333. doi: 10.1371/journal.pone.0119333 (PMC4356593; doi:10.1371/journal.pone.0119333)
Supplement: S6 Table — Results for the top 100 hits for “BMI longitudinal” GWAS analysis in GenABEL using each individual BMI reading as a separate observation, modelling the correlation between readings via the estimated kinship and using the genomic control deflation factor to avoid inflation of the overall distribution of test statistics. Results are for allele-wise tests under an additive model of inheritance. Bold indicates 3 top SNP associations for imputed data (P <10-6) coincide with two top genes of functional interest (NTRK2, PIK3C2G) presented in main Table 1, as well as a hit near DICER a previously observed GWAS hit for BMI (see S2 Table). (PDF) [file pone.0119333.s015.pdf]

Table S6. Top GWAS imputed SNP hits for BMI, organised by chromosome. Results for the top 100 hits for "BMI longitudinal" GWAS analysis in GenABEL using each individual BMI reading as a separate observation, modelling the correlation between readings via the estimated kinship and using the genomic control deflation factor to avoid inflation of the overall distribution of test statistics. Results are for allele-wise tests under an additive model of inheritance adjusting for sex and age[5]. Bold indicates 3 top SNP associations for imputed data ($P < 10^{-6}$) coincide with two top genes of functional interest (*NTRK2* , *PIK3C2G*) presented in main Table 1, as well as a hit near *DICER* a previously observed GWAS hit for BMI (see Table S2).

Chromosome	SNP	NCBI37	A1	A2	effB	se_effB	$P_{deflated}$	SNP Location	HGNC*
2	rs186440540	171505976	1	2	-0.42	0.10	1.88E-05	intron_variant	MYO3B
2	rs6710748	206738804	2	1	-0.44	0.10	1.56E-05	intergenic_variant	NRP2/INO80D
4	rs28580297	119427957	1	2	-1.46	0.33	8.57E-06	splice_acceptor_variant	CEP170P1
5	rs10074571	89845427	1	2	1.51	0.35	1.93E-05	intron_variant	GPR98
5	rs115347335	89846367	1	2	1.51	0.35	1.97E-05	intron_variant	GPR98
7	rs10235923	90788499	1	2	-0.39	0.09	1.56E-05	intron_variant	CDK14
7	rs10235925	90788503	1	2	-0.39	0.09	1.56E-05	intron_variant	CDK14
7	rs7786765	93199928	1	2	0.41	0.09	8.75E-06	intron_variant	CALCR
9	rs7871278	87135644	2	1	0.42	0.09	4.61E-06	intergenic_variant	SLC28A3/NTRK2
9	rs6559812	87139591	2	1	0.43	0.09	5.46E-06	intergenic_variant	SLC28A3/NTRK2
9	rs6559813	87139623	2	1	0.42	0.09	8.88E-06	intergenic_variant	SLC28A3/NTRK2
9	rs6559814	87140149	2	1	0.42	0.09	8.88E-06	intergenic_variant	SLC28A3/NTRK2
9	rs7028420	87140669	2	1	0.41	0.09	1.77E-05	intergenic_variant	SLC28A3/NTRK2
9	rs13299407	87141193	2	1	0.42	0.09	8.88E-06	intergenic_variant	SLC28A3/NTRK2
9	rs10868178	87142619	2	1	0.43	0.09	5.91E-06	intergenic_variant	SLC28A3/NTRK2
9	rs10868181	87142961	2	1	0.42	0.09	4.26E-06	intergenic_variant	SLC28A3/NTRK2
9	rs1926745	87143311	2	1	0.44	0.09	1.78E-06	intergenic_variant	SLC28A3/NTRK2
9	rs1926746	87143351	2	1	0.44	0.09	3.21E-06	intergenic_variant	SLC28A3/NTRK2
9	rs1926747	87143384	2	1	0.44	0.09	1.78E-06	intergenic_variant	SLC28A3/NTRK2
9	rs199828151	87143704	2	1	0.44	0.09	1.78E-06	intergenic_variant	SLC28A3/NTRK2
9	rs7857394	87144800	2	1	0.44	0.09	1.78E-06	intergenic_variant	SLC28A3/NTRK2
9	rs11140645	87145196	2	1	0.44	0.09	1.85E-06	intergenic_variant	SLC28A3/NTRK2
9	rs12352430	87146089	2	1	0.43	0.09	5.69E-06	intergenic_variant	SLC28A3/NTRK2
9	rs117281313	87146935	2	1	0.43	0.09	3.91E-06	intergenic_variant	SLC28A3/NTRK2
9	rs141458266	87147221	2	1	0.43	0.09	3.56E-06	intergenic_variant	SLC28A3/NTRK2
9	rs116623964	87147901	2	1	0.44	0.09	1.80E-06	intergenic_variant	SLC28A3/NTRK2
9	rs10217214	87148954	2	1	0.42	0.09	8.70E-06	intergenic_variant	SLC28A3/NTRK2
9	rs10217219	87149090	2	1	0.44	0.09	3.19E-06	intergenic_variant	SLC28A3/NTRK2
9	rs6559815	87149095	2	1	0.44	0.09	1.80E-06	intergenic_variant	SLC28A3/NTRK2
9	rs9696613	87149503	2	1	0.43	0.09	4.16E-06	intergenic_variant	SLC28A3/NTRK2
9	rs7027550	87150853	2	1	0.44	0.09	2.79E-06	intergenic_variant	SLC28A3/NTRK2
9	rs10217324	87151162	2	1	0.44	0.09	1.80E-06	intergenic_variant	SLC28A3/NTRK2
9	rs10868187	87151680	2	1	0.44	0.09	3.19E-06	intergenic_variant	SLC28A3/NTRK2
9	rs4877862	87151766	2	1	0.44	0.09	1.80E-06	intergenic_variant	SLC28A3/NTRK2
**9**	**rs11140653**	**87152509**	**2**	**1**	**0.48**	**0.09**	**2.90E-07**	**intergenic_variant**	**SLC28A3/NTRK2**
9	rs10868188	87153069	2	1	0.44	0.09	3.19E-06	intergenic_variant	SLC28A3/NTRK2
9	chr9:87153416:I	87153416	2	1	0.44	0.09	1.80E-06	intergenic_variant	SLC28A3/NTRK2
9	rs11140654	87154451	2	1	0.44	0.09	1.80E-06	intergenic_variant	SLC28A3/NTRK2
9	rs11140655	87154661	2	1	0.44	0.09	1.80E-06	intergenic_variant	SLC28A3/NTRK2
9	rs10868189	87155482	2	1	0.44	0.09	3.19E-06	intergenic_variant	SLC28A3/NTRK2
9	rs7875895	87155579	2	1	0.44	0.09	1.80E-06	intergenic_variant	SLC28A3/NTRK2
9	rs10746743	87157630	2	1	0.44	0.09	1.80E-06	intergenic_variant	SLC28A3/NTRK2
9	rs10868190	87158048	2	1	0.40	0.09	1.85E-05	intergenic_variant	SLC28A3/NTRK2
9	rs10746744	87158856	2	1	0.40	0.09	1.85E-05	intergenic_variant	SLC28A3/NTRK2
9	rs12684594	87159019	2	1	0.44	0.09	3.24E-06	intergenic_variant	SLC28A3/NTRK2
9	rs4877278	87159864	2	1	0.44	0.09	1.80E-06	intergenic_variant	SLC28A3/NTRK2
9	rs10868191	87160278	2	1	0.44	0.09	2.27E-06	intergenic_variant	SLC28A3/NTRK2
9	rs10780680	87160754	2	1	0.44	0.09	1.80E-06	intergenic_variant	SLC28A3/NTRK2
9	rs10868192	87161646	2	1	0.40	0.09	1.85E-05	intergenic_variant	SLC28A3/NTRK2
9	rs11140658	87162029	2	1	0.41	0.10	1.37E-05	intergenic_variant	SLC28A3/NTRK2
9	rs11140659	87162361	2	1	0.44	0.09	1.80E-06	intergenic_variant	SLC28A3/NTRK2
9	rs5898864	87164995	2	1	0.44	0.09	3.24E-06	intergenic_variant	SLC28A3/NTRK2
9	rs202216063	87165309	2	1	0.42	0.10	1.16E-05	intergenic_variant	SLC28A3/NTRK2
9	rs12338461	87165386	2	1	0.40	0.09	1.85E-05	intergenic_variant	SLC28A3/NTRK2
9	rs7039054	87165771	2	1	0.44	0.09	1.80E-06	intergenic_variant	SLC28A3/NTRK2
9	rs11140662	87166608	2	1	0.44	0.09	1.80E-06	intergenic_variant	SLC28A3/NTRK2
9	rs2068225	87168094	2	1	0.44	0.09	3.20E-06	intergenic_variant	SLC28A3/NTRK2
9	rs1992242	87169606	2	1	0.43	0.09	3.34E-06	intergenic_variant	SLC28A3/NTRK2

Supplementary Table 6

Chromosome	SNP	NCBI37	A1	A2	effB	se_effB	$P_{deflated}$	SNP Location	HGNC*
9	rs4877864	87170731	2	1	0.45	0.09	1.67E-06	intergenic_variant	SLC28A3/NTRK2
9	rs1439051	87171338	2	1	0.45	0.09	1.67E-06	intergenic_variant	SLC28A3/NTRK2
9	rs1347856	87173092	2	1	0.44	0.09	3.74E-06	intergenic_variant	SLC28A3/NTRK2
9	rs1347857	87173097	2	1	0.44	0.09	2.09E-06	intergenic_variant	SLC28A3/NTRK2
9	rs1347858	87173333	2	1	0.44	0.09	3.74E-06	intergenic_variant	SLC28A3/NTRK2
9	rs1439053	87177321	2	1	0.44	0.09	2.09E-06	intergenic_variant	SLC28A3/NTRK2
9	rs10868193	87178426	2	1	0.44	0.09	2.09E-06	intergenic_variant	SLC28A3/NTRK2
9	rs1866439	87179264	2	1	0.44	0.09	2.09E-06	intergenic_variant	SLC28A3/NTRK2
9	rs10868194	87179908	2	1	0.44	0.09	2.09E-06	intergenic_variant	SLC28A3/NTRK2
9	rs7858448	87180554	2	1	0.44	0.09	3.74E-06	intergenic_variant	SLC28A3/NTRK2
9	rs10868200	87185183	2	1	0.45	0.10	3.74E-06	intergenic_variant	SLC28A3/NTRK2
9	rs10868201	87185249	2	1	0.42	0.09	7.94E-06	intergenic_variant	SLC28A3/NTRK2
9	rs4877280	87186405	2	1	0.42	0.09	7.21E-06	intergenic_variant	SLC28A3/NTRK2
9	rs11140671	87188675	2	1	0.42	0.09	8.86E-06	intergenic_variant	SLC28A3/NTRK2
9	rs11140675	87189619	2	1	0.44	0.09	2.27E-06	intergenic_variant	SLC28A3/NTRK2
9	rs10868204	87189655	2	1	0.43	0.09	4.34E-06	intergenic_variant	SLC28A3/NTRK2
9	rs10868207	87190559	2	1	0.44	0.09	2.27E-06	intergenic_variant	SLC28A3/NTRK2
9	rs2889960	87193464	2	1	0.43	0.10	1.06E-05	intergenic_variant	SLC28A3/NTRK2
9	rs2118819	87193728	2	1	0.43	0.10	1.06E-05	intergenic_variant	SLC28A3/NTRK2
12	rs12810466	18769889	1	2	0.72	0.16	8.22E-06	intron_variant	PIK3C2G
12	rs12816270	18770577	1	2	0.72	0.16	8.69E-06	intron_variant	PIK3C2G
12	rs12813646	18775223	1	2	0.70	0.16	1.31E-05	intron_variant	PIK3C2G
12	rs35801168	18778311	1	2	0.71	0.16	8.63E-06	intron_variant	PIK3C2G
**12**	**rs10841048**	**18779259**	**1**	**2**	**0.71**	**0.14**	**6.28E-07**	**intron_variant**	**PIK3C2G**
13	rs12865097	45477410	1	2	0.78	0.17	6.83E-06	intergenic_variant	TSC22D1/NUFIP
**14**	**rs12897644**	**95838401**	**1**	**2**	**1.54**	**0.31**	**8.61E-07**	**intergenic_variant**	**DICER1,CLMN/SYNE3**
15	rs936674	93903597	2	1	-1.36	0.30	4.65E-06	intron_variant	RP11-266O8.1
17	rs8077139	40750099	2	1	-0.65	0.15	2.02E-05	intron_variant	FAM134C
18	rs453137	3490546	1	2	-1.08	0.24	9.67E-06	intergenic_variant	TGIF1/DLGAP1
18	rs1791221	29219891	2	1	-0.39	0.09	2.03E-05	intron_variant	B4GALT6
18	rs1667281	29220756	2	1	-0.39	0.09	2.03E-05	intron_variant	B4GALT6
18	rs1612691	29220918	2	1	-0.39	0.09	2.03E-05	intron_variant	B4GALT6
18	rs61735186	61471529	1	2	-1.33	0.30	1.31E-05	missense_variant	SERPINB7
18	rs35453062	61602360	1	2	-1.33	0.30	1.31E-05	missense_variant	SERPINB10
18	rs7229301	62312564	1	2	-1.33	0.30	1.08E-05	intergenic_variant	SERPINB8/CDH7
18	chr18:62325039:D	62325039	1	2	-1.33	0.30	1.08E-05	intergenic_variant	SERPINB8/CDH7
18	rs9945604	62325040	1	2	-1.33	0.30	1.08E-05	intergenic_variant	SERPINB8/CDH7
18	rs114050355	62340309	1	2	-1.33	0.30	1.08E-05	intergenic_variant	SERPINB8/CDH7
19	rs183353539	6992244	1	2	-1.37	0.31	8.19E-06	upstream_gene_variant	EMR4P
19	rs142674362	8809964	1	2	-1.60	0.37	1.88E-05	upstream_gene_variant	ACTL9
22	rs55769859	46353046	1	2	1.37	0.30	5.36E-06	intron_variant	WNT7B
22	rs117707689	46375471	1	2	1.92	0.43	7.37E-06	upstream_gene_variant	WNT7B
