# Supplementary material for: First Genome-Wide Association Study in an Australian Aboriginal Population Provides Insights into Genetic Risk Factors for Body Mass Index and Type 2 Diabetes
Source: PLoS One. 2015 Mar 11;10(3):e0119333. doi: 10.1371/journal.pone.0119333 (PMC4356593; doi:10.1371/journal.pone.0119333)
Supplement: S7 Table — Results are for the top 100 hits for T2D GWAS analysis in GenABEL for allele-wise tests under an additive model. Bold indicates 3 top SNP associations for imputed data (P<10-6) not observed in the genotyped data. Bold plus grey shading indicates hits that coincide with top gene of functional interest (BCL9) presented in main Table 2, as well as a hit near IGF2BP2 a previously observed GWAS hit for T2D (see S4 Table). (PDF) [file pone.0119333.s016.pdf]

Table S7. Top GWAS imputed SNP hits for T2D, organised by chromosome. Results are for the top 100 hits for T2D GWAS analysis in GenABEL for allele-wise tests under an additive model. Bold indicates 3 top SNP associations for imputed data ($P<10^{-6}$) not observed in the genotyped data. Bold plus grey shading indicates hits that coincide with top gene of functional interest (*BCL9*) presented in main Table 2, as well as a hit near *IGF2BP2* a previously observed GWAS hit for T2D (see Table S4).

Chr	SNP	NCBI37	A1	A2	effB	se_effB	P1df	SNP Location	HGNC
1	rs113059126	85081330	1	2	0.21	0.05	1.17E-05	intergenic_variant	CTBS/SSX2IP
1	rs78964768	85083271	1	2	0.21	0.05	1.12E-05	intergenic_variant	CTBS/SSX2IP
1	chr1:146995382:I	146995382	1	2	0.19	0.04	1.70E-05	intergenic_variant	CHD1L/BCL9
**1**	**rs11240074**	**146996480**	**1**	**2**	**0.21**	**0.05**	**5.73E-06**	**intergenic_variant**	**CHD1L/BCL9**
**1**	**rs11240075**	**146996800**	**1**	**2**	**0.21**	**0.05**	**5.73E-06**	**intergenic_variant**	**CHD1L/BCL9**
1	rs114304204	180195390	1	2	0.60	0.14	1.34E-05	upstream_gene_variant	LHX4
2	rs13416871	37634382	C	T	0.56	0.13	1.34E-05	intergenic_variant	QPCT/CDC42EP3
2	rs189473206	37646924	1	2	0.56	0.13	1.28E-05	intergenic_variant	QPCT/CDC42EP3
2	rs74715015	37992445	1	2	0.62	0.14	1.15E-05	intergenic_variant	CDC42EP3/RMDN2
2	chr2:40524173:I	40524173	1	2	0.57	0.13	1.47E-05	intron_variant	SLC8A1
2	rs115376879	40532999	A	T	0.62	0.14	1.43E-05	intron_variant	SLC8A1
2	chr2:40535118:D	40535118	1	2	0.62	0.14	1.43E-05	intron_variant	SLC8A1
2	chr2:40535131:D	40535131	1	2	0.62	0.14	1.43E-05	intron_variant	SLC8A1
2	rs115465855	40536118	1	2	0.62	0.14	1.43E-05	intron_variant	SLC8A1
2	rs114204321	40537878	1	2	0.62	0.14	1.43E-05	intron_variant	SLC8A1
2	rs116518660	40540379	1	2	0.62	0.14	1.43E-05	intron_variant	SLC8A1
2	rs116379507	40540840	1	2	0.62	0.14	1.43E-05	intron_variant	SLC8A1
2	rs114252186	40541632	1	2	0.62	0.14	1.43E-05	intron_variant	SLC8A1
2	rs114890647	40541709	1	2	0.62	0.14	1.43E-05	intron_variant	SLC8A1
2	rs80068953	40542303	1	2	0.62	0.14	1.43E-05	intron_variant	SLC8A1
2	rs114360751	40542486	1	2	0.62	0.14	1.43E-05	intron_variant	SLC8A1
2	rs114553436	40545570	1	2	0.62	0.14	1.43E-05	intron_variant	SLC8A1
2	rs115067949	40547285	1	2	0.62	0.14	1.43E-05	intron_variant	SLC8A1
2	rs6751854	40547861	1	2	0.62	0.14	1.43E-05	intron_variant	SLC8A1
2	rs115114409	40548573	1	2	0.62	0.14	1.43E-05	intron_variant	SLC8A1
2	rs114290845	40548946	1	2	0.62	0.14	1.43E-05	intron_variant	SLC8A1
2	rs140493875	40550246	1	2	0.62	0.14	1.43E-05	intron_variant	SLC8A1
2	rs114635799	40550250	1	2	0.62	0.14	1.43E-05	intron_variant	SLC8A1
2	rs72943133	40550699	1	2	0.62	0.14	1.43E-05	intron_variant	SLC8A1
2	rs34669198	45748277	1	2	0.30	0.06	1.35E-06	intron_variant	SRBD1
2	rs3770284	45760123	1	2	0.30	0.06	1.81E-06	intron_variant	SRBD1
2	rs3770289	45764951	1	2	0.30	0.06	1.81E-06	intron_variant	SRBD1
2	rs755654	45770222	1	2	0.31	0.07	2.20E-06	intron_variant	SRBD1
2	rs116393692	199284836	A	G	0.36	0.08	1.50E-05	intron_variant	PLCL1
2	rs190834432	200552496	1	2	0.37	0.08	1.20E-06	intergenic_variant	SATB2/TYW5
**2**	**chr2:200603251:I**	**200603251**	**1**	**2**	**0.38**	**0.07**	**1.19E-07**	**intergenic_variant**	**SATB2/TYW5**
2	rs75260059	200616257	1	2	0.34	0.07	8.97E-07	intergenic_variant	SATB2/TYW5
2	rs116779211	200620233	1	2	0.34	0.07	8.97E-07	intergenic_variant	SATB2/TYW5
2	rs58158314	232279412	1	2	0.67	0.15	1.57E-05	intergenic_variant	B3GNT7/ZBTB8OSP2
3	rs114015970	64061521	C	G	0.60	0.14	1.54E-05	intergenic_variant	PSMD6/PRICKLE2
3	rs12496230	66829908	T	C	0.54	0.12	4.26E-06	intergenic_variant	LRIG1/KBTBD8
3	rs13100186	66831642	1	2	0.54	0.12	4.26E-06	intergenic_variant	LRIG1/KBTBD8
3	rs17782285	66833592	1	2	0.54	0.12	4.26E-06	intergenic_variant	LRIG1/KBTBD8
3	rs34508066	66837509	1	2	0.54	0.12	4.26E-06	intergenic_variant	LRIG1/KBTBD8
3	rs36016775	66838437	1	2	0.54	0.12	4.26E-06	intergenic_variant	LRIG1/KBTBD8
3	rs13079612	66840534	1	2	0.54	0.12	4.26E-06	intergenic_variant	LRIG1/KBTBD8
3	rs4856960	66843301	1	2	0.54	0.12	4.26E-06	intergenic_variant	LRIG1/KBTBD8
3	rs1036972	66846010	1	2	0.54	0.12	4.26E-06	intergenic_variant	LRIG1/KBTBD8
3	rs13096599	66848809	1	2	0.54	0.12	4.26E-06	intergenic_variant	LRIG1/KBTBD8
3	rs62258925	66849747	1	2	0.54	0.12	4.26E-06	intergenic_variant	LRIG1/KBTBD8
3	chr3:66850670:D	66850670	1	2	0.53	0.12	4.65E-06	intergenic_variant	LRIG1/KBTBD8
3	chr3:66850676:D	66850676	1	2	0.53	0.12	4.65E-06	intergenic_variant	LRIG1/KBTBD8
3	rs1036971	66850677	1	2	0.53	0.12	4.65E-06	intergenic_variant	LRIG1/KBTBD8
3	rs1036970	66850707	1	2	0.53	0.12	4.65E-06	intergenic_variant	LRIG1/KBTBD8
3	rs34954762	66853890	1	2	0.54	0.12	4.26E-06	intergenic_variant	LRIG1/KBTBD8
3	rs3899502	66856411	1	2	0.54	0.12	4.26E-06	intergenic_variant	LRIG1/KBTBD8
3	rs12495879	66861656	1	2	0.54	0.12	4.26E-06	intergenic_variant	LRIG1/KBTBD8
3	rs62258957	66864153	1	2	0.54	0.12	4.26E-06	intergenic_variant	LRIG1/KBTBD8

Supplementary Table 7

Chr	SNP	NCBI37	A1	A2	effB	se_effB	P1df	SNP Location	HGNC
3	rs4856828	66868008	1	2	0.54	0.12	4.26E-06	intergenic_variant	LRIG1/KBTBD8
3	rs12494775	66874105	1	2	0.53	0.12	4.38E-06	intergenic_variant	LRIG1/KBTBD8
**3**	**rs138306797**	**185545719**	**1**	**2**	**0.78**	**0.16**	**2.55E-06**	**upstream_gene_variant**	**IGF2BP2**
3	rs148055746	187530476	C	T	0.60	0.14	1.46E-05	intergenic_variant	BCL6/LPP
3	rs149049329	187597927	1	2	0.71	0.15	1.87E-06	intergenic_variant	BCL6/LPP
4	rs11728703	40024341	C	G	-0.16	0.04	1.13E-05	intergenic_variant	PD55A/N4BP2
4	rs114542811	125202226	C	T	0.60	0.14	8.68E-06	intergenic_variant	-
4	rs10518429	125213499	1	2	0.60	0.14	8.81E-06	intergenic_variant	-
5	rs4703911	82012854	C	T	0.22	0.05	8.73E-06	intergenic_variant	ATP6AP1L/TMEM167A
5	rs1423508	82014270	2	1	0.22	0.05	8.78E-06	intergenic_variant	ATP6AP1L/TMEM167A
6	rs149552992	108566395	A	G	0.43	0.10	1.61E-05	intron_variant	SNX3
6	chr6:113697599:I	113697599	1	2	0.58	0.13	4.77E-06	intergenic_variant	SOCS5P5/MARCKS
6	rs148167105	113728568	T	A	0.57	0.13	6.06E-06	intergenic_variant	SOCS5P5/MARCKS
6	rs79891214	113754685	1	2	0.58	0.13	4.79E-06	intergenic_variant	SOCS5P5/MARCKS
6	rs148230200	113757021	1	2	0.58	0.13	4.79E-06	intergenic_variant	SOCS5P5/MARCKS
6	rs183165668	113872189	1	2	0.58	0.13	4.57E-06	intergenic_variant	SOCS5P5/MARCKS
6	rs149358103	113908049	1	2	0.58	0.13	4.70E-06	intergenic_variant	SOCS5P5/MARCKS
6	rs77749793	113962810	1	2	0.58	0.13	4.70E-06	intergenic_variant	SOCS5P5/MARCKS
6	chr6:114183873:D	114183873	1	2	0.57	0.13	5.37E-06	intergenic_variant	SOCS5P5/MARCKS
6	rs58261156	151451109	C	T	0.60	0.14	1.18E-05	intergenic_variant	MTHFD1L/AKAP12
6	rs7756756	151453165	1	2	0.60	0.14	1.18E-05	intergenic_variant	MTHFD1L/AKAP12
6	rs7762382	151454344	1	2	0.60	0.14	1.18E-05	intergenic_variant	MTHFD1L/AKAP12
6	rs57158969	151455324	1	2	0.60	0.14	1.18E-05	intergenic_variant	MTHFD1L/AKAP12
**6**	**rs9478961**	**151456593**	**1**	**2**	**0.44**	**0.08**	**1.80E-07**	**intergenic_variant**	**MTHFD1L/AKAP12**
7	rs7794890	22412741	2	1	0.15	0.03	9.80E-06	intergenic_variant	RAPGEF5/STEAP1B
7	rs78598078	83461593	1	2	0.47	0.11	1.69E-05	intergenic_variant	SEMA3E/SEMA3A
12	rs7978895	43570515	A	T	0.14	0.03	9.08E-06	intergenic_variant	/ADAMTS20
12	rs1849784	43572374	A	G	0.14	0.03	1.26E-05	intergenic_variant	/ADAMTS20
13	rs76176894	45782642	T	C	0.29	0.07	9.19E-06	intron_variant	GTF2F2
13	chr13:45823954:I	45823954	1	2	0.30	0.06	2.76E-06	intron_variant	GTF2F2
13	rs76661672	45844847	1	2	0.28	0.07	1.54E-05	intron_variant	GTF2F2
13	rs146140864	45859647	1	2	0.30	0.06	2.76E-06	intron_variant	GTF2F2
13	rs79490558	45886186	C	A	0.31	0.07	1.36E-06	intergenic_variant	GTF2F2/TPT1
13	rs78319313	71595310	1	2	0.49	0.11	2.84E-06	intron_variant	LINC00348
**13**	**rs138128600**	**73764625**	**T**	**G**	**0.53**	**0.10**	**1.55E-07**	intergenic_variant	**KLF5/KLF12**
13	rs144576319	73789048	1	2	0.41	0.09	7.72E-06	intergenic_variant	KLF5/KLF12
14	rs115174845	22207842	1	2	0.63	0.14	1.12E-05	downstream_gene_variant	TRAV4
16	rs189178161	12718853	T	A	0.64	0.14	8.10E-06	intergenic_variant	SNX29/,CPPED1
16	chr16:12721359:I	12721359	1	2	0.64	0.14	6.33E-06	intergenic_variant	SNX29/,CPPED1
18	rs6506284	5030023	1	2	0.32	0.07	3.29E-06	intergenic_variant	/C18orf42
19	rs184399074	9153577	1	2	0.51	0.12	1.37E-05	intergenic_variant	MUC16/OR1M1
20	rs13036344	31313737	1	2	0.20	0.04	9.48E-06	intron_variant	COMMD7
