# Supplementary material for: First Genome-Wide Association Study in an Australian Aboriginal Population Provides Insights into Genetic Risk Factors for Body Mass Index and Type 2 Diabetes
Source: PLoS One. 2015 Mar 11;10(3):e0119333. doi: 10.1371/journal.pone.0119333 (PMC4356593; doi:10.1371/journal.pone.0119333)
Supplement: S1 Text — (PDF) [file pone.0119333.s017.pdf]

S1 Text. Supplement on Conditional Analysis

Analysis of T2D and the standardised BMI residual (BMI-long) was initially performed without any covariate adjustment. Given the expected relationship between BMI and the development of T2D, we repeated these analyses allowing for covariates as follows:

Analyses of T2D

We performed a GWAS of T2D, allowing for mean standardised BMI residual (averaged over the repeated BMI readings) as a covariate. This analysis effectively searches for genetic associations with T2D operating independently of the standardised BMI residual (which is the BMI-related variable that we interrogated through GWAS).

One disadvantage of this analysis is loss of power compared to our original T2D analysis, as only the 361 individuals with BMI readings (excluding the BMI outlier) out of the original 391 individuals with known T2D status contribute to the final analysis.

The plot below shows (A. Genotyped GWAS analysis; B. Imputed GWAS analysis) the $-\log_{10}$ *P*-values from the original T2D analysis (no covariate adjustment) and the analysis adjusted for mean standardised BMI residual plotted against one another (with each point representing the *P*-values from the 2 analyses being compared, for a given SNP).

(A) Genotyped GWAS

(B) Imputed GWAS

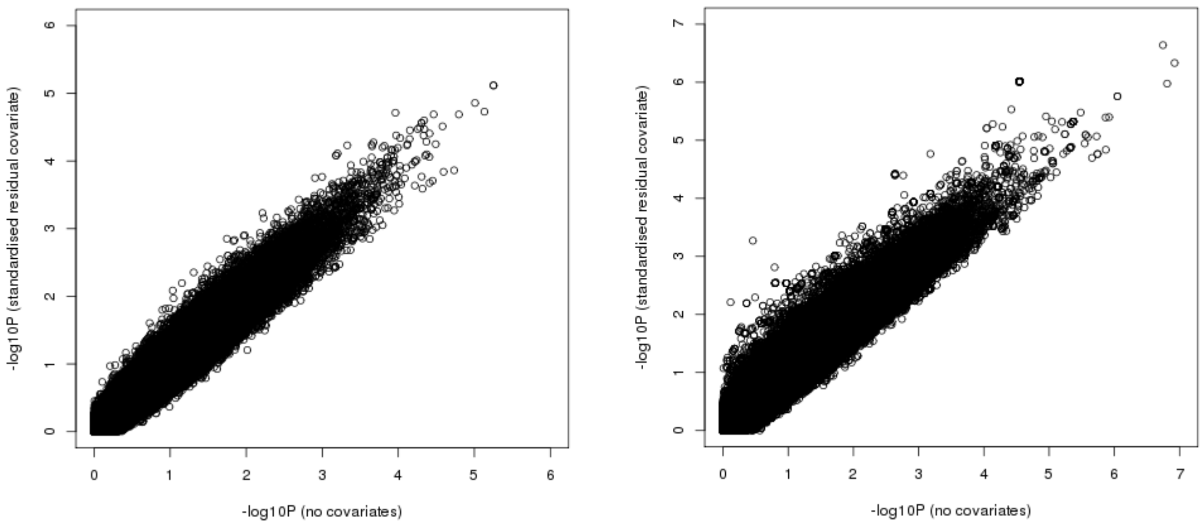

It can be seen that the results when including an adjustment for mean standardised BMI residual are highly correlated with the original results. This suggests that genetic effects on T2D as originally calculated are largely independent of any effects attributable to the mean standardised BMI residual which essentially measures the BMI value *over and above* what would be expected from the general population value for an individual of a given age and sex – an expected value that, in this population, most likely already includes the effect of diet and other environmental influences, and thus (indirectly) accounts for population-level raw BMI.

Given that the majority of our SNPs do not, in any case, reach the standard for declaring genome-wide statistical significance, we would be wary of over-interpretation of these results for specific individual SNPs. However, we note (table below) that, for the top SNPs ($P<10^{-5}$) in genes of functional significance originally reported in main Table 2, the results remain significant regardless of covariate adjustment:

S1 Text. Supplement on Conditional Analysis

Results when mean standardised BMI residual is included as a covariate:

Conditional analysis for top GWAS SNP hits in genes of functional interest for T2D, organised by chromosome.													
					Genotyped				Imputed 1000G					
Chromosome	SNP	NCBI37	A1	A2	effB	se_effB	chi2.1df	P1df	effB	se_effB	chi2.1df	P1df	SNP Location	HGNC*
**1**	**rs11240074**	**146996480**	**A**	**C**	**0.22**	**0.05**	**20.02**	**7.66E-06**					**INTERGENIC**	**CHD1L/BCL9**
	**rs11240074**	**146996480**	**A**	**C**					**0.22**	**0.05**	**19.98**	**7.83E-06**	**INTERGENIC**	**CHD1L/BCL9**
6	rs6930407	89905239	A	G	0.14	0.03	17.19	3.38E-05					INTRONIC	GABRR1
	rs9451177	89905172	A	G					0.14	0.03	18.24	1.95E-05	INTRONIC	GABRR1
12	rs11063387	4998536	G	C	0.14	0.04	14.02	1.80E-04					INTERGENIC	KCNA6/KCNA1
	rs11063385	4997051	C	T					0.13	0.04	13.57	2.30E-04	INTERGENIC	KCNA6/KCNA1
21	rs8128418	39188732	G	A	0.24	0.07	13.13	2.90E-04					INTRONIC	KCNJ6
	rs113713721	39185153	G	A					0.25	0.07	13.97	1.85E-04	INTRONIC	KCNJ6

* Genes separated by forward slash indicate nearest protein coding genes upstream/downstream of the SNP. NCBI37 = bp location on chromosome for NCBI Build 37. A1 = major allele; A2 = minor allele. Bold indicates top hit for T2D based on both genotyped and imputed data.

Analyses of BMI

We repeated our GWAS for the standardised BMI residual (BMI-long) using T2D (which effectively corresponds to whether someone was ever diagnosed with T2D) as a covariate. Given that the original results from GenABEL and FaST-LMM were not completely concordant (although highly correlated, see **Figure S4A**) we performed this analysis separately in both packages. For both GenABEL and Fast-LMM, the concordance in –log10 *P*-values with/without including T2D as a covariate is extremely high.

(A) GenABEL (B) Fast-LMM

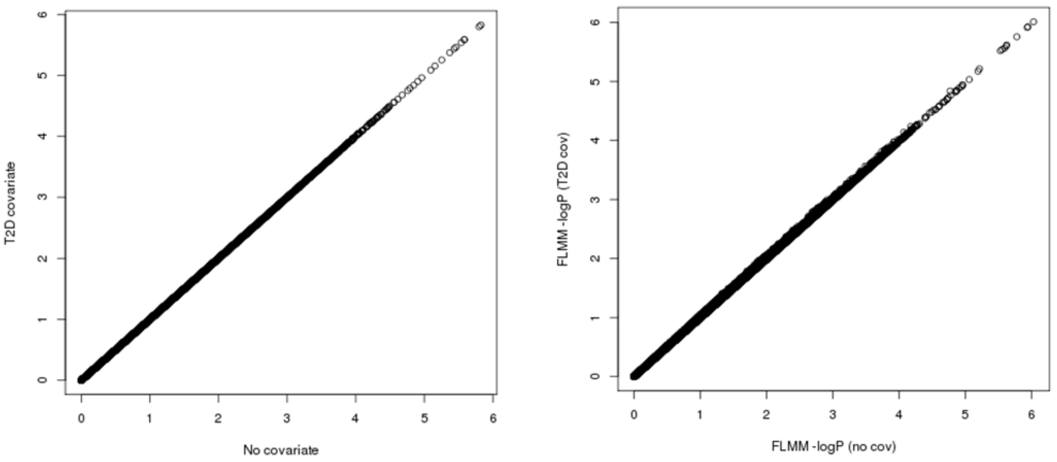

Thus it seems that the genetic effects contributing to the standardised BMI residual as originally calculated are operating independent of any T2D effect. This is consistent with regional association plots shown in **Supplementary Figure 7** where no evidence for association is seen in plots comparing T2D results across regions that contain the top hits for BMI. Given the extremely strong concordances observed above when using the GWAS data, we did not repeat this analysis using the imputed data.
